# The inhibitive action of 2-mercaptobenzothiazole on the porosity of corrosion film formed on aluminum and aluminum–titanium alloys in hydrochloric acid solution

**DOI:** 10.1038/s41598-023-31795-2

**Published:** 2023-03-23

**Authors:** Abdel-Rahman El-Sayed, Morad M. El-Hendawy, Mohamed Sarwat El-Mahdy, Fatma S. M. Hassan, Adila E. Mohamed

**Affiliations:** 1 Department of Chemistry, Faculty of Science, Sohag University, Sohag, 82524 Egypt; 2 Department of Chemistry, Faculty of Science, New Valley University, Kharga, 72511 Egypt; 3 Department of Chemistry, Faculty of Science, Aswan University, Aswan, 81528 Egypt

**Keywords:** Chemistry, Energy

## Abstract

2-Mercaptobenzothiazole (2-MBT) in a solution of 0.5 M HCl is an effective corrosion inhibitor for aluminum and aluminum–titanium alloys. Tafel polarization and electrochemical impedance spectroscopy (EIS) were employed to assess this heterocyclic compound’s anticorrosive potential and complementary by scanning electron microscope (SEM) and calculating porosity percentage in the absence and presence of various inhibitor concentrations. Inhibition efficiency (*IE*%) was strongly related to concentration (10^–6^–10^–3^ M). Temperature’s effect on corrosion behavior was investigated. The data exhibited that the *IE*% decreases as the temperature increases. An increase in activation energy (*E*_a_) with increasing the inhibitor concentration and the decrease in the *IE*% value of the mentioned compound with raising the temperature indicates that the inhibitor molecules are adsorbed physically on the surface. Thermodynamic activation parameters for Al and Al–Ti alloy dissolution in both 0.5 M HCl and the inhibited solution were calculated and discussed. According to Langmuir’s adsorption isotherm, the inhibitor molecules were adsorbed. The evaluated standard values of the enthalpy ($\Delta {H}_{ads.}^{o})$, entropy ($\Delta {S}_{ads.}^{o})$ and free energy changes ($\Delta {G}_{ads.}^{o})$ showed that $\Delta {H}_{ads.}^{o}$ and $\Delta {G}_{ads.}^{o}$ are negative, while $\Delta {S}_{ads.}^{o}$ was positive. The formation of a protective layer adsorbed on the surfaces of the substrates was confirmed with the surface analysis (SEM). The porosity percentage is significantly reduced in the inhibitor presence and gradually decreased with increasing concentration. Furthermore, the density functional theory (DFT) and Monte Carlo (MC) simulations were employed to explain the variance in protecting the Al surface from corrosion. Interestingly, the theoretical findings align with their experimental counterparts. The planarity of 2-MBT and the presence of heteroatoms are the playmakers in the adsorption process.

## Introduction

For decades, lightweight alloys based on aluminum (Al) and titanium (Ti) have been debated and researched. The Al–Ti binary system’s resultant alloys with hard intermetallic phases typically show good microstructural and mechanical characteristics^1^. Al–Ti alloy is characterized by low densities, high specific strength, and reduced manufacturing cost. Aluminum alloys are frequently used in aircraft because of their high strength to weigh ratio^2^. Adding Ti to Al increases the material strength, but the presence of Ti in the Al–Ti alloy increases the corrosion rate compared with pure Al. Owing to the generation of intermetallic phase on the Al–Ti alloy surface, it is more susceptible to corrosive attacks^1^. Therefore, the presence of Al_3_Ti phases in the alloy can be catalyzed via chemical and electrochemical processes on the alloy surface. For this reason, working anodes and cathodes contribute to matrix dealloying^3^. However, the protective coating of Al_2_O_3_ that forms on the surface appears chemically unstable in both alkaline and acidic conditions. Subsequently, these environments are harmful to aluminum and its alloys^4^.

It is well known that Al and its alloys showed a high corrosion rate in acidic solutions. HCl is used to etch Al and its alloys chemically and electrochemically. Also, HCl solution is used to remove the oxides forming on the surfaces. Therefore, the corrosion rates of the investigated metal and its alloys should be retarded using organic inhibitors during the etching process. The corrosion processes were inhibited by the organic additives introduced to the acidic solution is due to the surface adsorption of the inhibitor molecules^3,5^. This adsorption occurs by physical adsorption through electrostatic action between the surface and organic molecules. However, chemisorption occurs by forming coordination bonds between the surface and inhibitor molecules. These studies aim to prevent the metal or alloy from a corrosive medium^5^. The first inhibiting mechanism in HCl solution is the organic compounds’ adsorption on the surface of metal or alloy. In order to explain the passive oxide layer breakdown that occurs when Cl^−^ ions reach the film, many mechanisms have been mentioned^6,7^. One of such mechanism explains that Cl^−^ ions may not enter the oxide layer; instead, they are chemisorbed on this layer^5^.

Generally, the selection of organic inhibitors containing sulfur, nitrogen and/or oxygen as polar groups, which are connected with double bonds in their compositions, have been recognized as good corrosion inhibitors for various alloys and metals in acidic solutions^8–11^. Some factors play an essential role in the adsorption process on the surface of metal or alloy, such as the nature and the type of charge on the surface, the type of the studied solution, and the chemical structure of the organic compound^12^.

Aluminum exposed to acidic or alkaline solutions should be treated with an efficient corrosion inhibitor. Corrosion inhibitor procedures are defined as either cathodic, anodic, or mixed inhibition based on their capacity to slow metal dissolution and reduction. Organic inhibitors generally have a dissimilar influence on both the cathodic and anodic processes^13–15^. The corrosion inhibition efficiency of imidazolium-based inhibitors in H_2_SO_4_ on mild steel was explored experimentally and theoretically^16^. The anticorrosive capabilities of benzimidazole and its derivatives are due to the π-electrons on the planar-fused moiety and the lone pair of electrons on the hetero-atoms. Using DFT and molecular dynamics simulations, Obot et al.^17^ investigated the adsorption mechanism of 2-mercaptobenzimidazole (2-MBI) as a corrosion inhibitor for Fe (110), Cu (111), and Al (111) surfaces. The data shows the higher anticorrosive performance of 2-MBI on steel corrosion compared to aluminum and copper.

Several investigations have already been conducted on the corrosion inhibition properties of 2-mercaptobenzothiazole (2-MBT) on AA6082 alloy, Cu, C-steel, and AA 2024-T3 alloy (Cu-rich intermetallic particles)^6,18–20^. The results of these studies show that 2-MBT can operate as a good corrosion inhibitor. However, due to the diversity of organic molecules, producing a highly efficient inhibitor faces significant obstacles. An extensive review of the relevant documented sources confirms that 2-mercaptobenzothiazole was not previously been used as a corrosion inhibitor for Al and Al–Ti alloy.

This work aims to introduce an electrochemical study of the new effect of (2-MBT) on corrosion inhibition of aluminum and aluminum–titanium alloys in HCl (0.5 M) solutions using Tafel polarization and electrochemical impedance spectroscopy (EIS) techniques at various concentrations and temperatures, as well as a scanning electron microscope (SEM) for surface characterization. On the other hand, the DFT and MC approaches were applied to relate the detected inhibition efficiency associated with the quantum chemical descriptors of the studied inhibitor and the adsorption parameters of the inhibitor/Al(111) complexes. Correspondingly, the porosity percentage on the surfaces of Al and Al–Ti alloy was evaluated using both SEM micrographs and Tafel polarization data in both the absence and presence of the tested inhibitor.

## Experimental procedures

### Materials and solutions

Al and Ti were the starting components, both being 99.99% pure. Al ingots were blasted to a temperature of more than 660 °C before various amounts of Ti (0, 1, 2, 5, and 8 wt%) were supplied. The molten solution was elevated to a pouring temperature (900–1400 °C) using a 200-kW medium frequency induction furnace (type ABB, Germany) with protective Ar gas after each Ti addition. Finally, each alloy melt was poured into a worm-shaped cast iron mold. The morphology and composition of the studied alloys have been assessed utilizing SEM and X-ray diffraction. The homogeneous composition of the solid solution phase was found to exist^1^.

**Figure 1:**
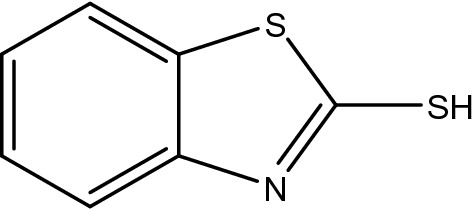
Structure of 2-mercaptobenzothiazole (2-MBT).

Table 1 shows the compositions of Al and Al–Ti alloys. 2-Mercaptobenzothiazole (2-MBT) was obtained from Alfa Aesar (Fig. 1). It was employed at quantities ranging from 1 × 10^−6^ to 1 × 10^−3^ M without further purification. Diluting AR grade HCl with water yielded the corrosive solution (0.5 M HCl).

**Table 1:** Wt% of the prepared Al–Ti alloy.

Alloy	Al	AT-1	AT-2	AT-3	AT-4
Wt%	Al_100_Ti_0_	Al_98.7_Ti_1.3_	Al_98.27_Ti_1.73_	Al_94.7_Ti_5.3_	Al_91.27_Ti_8.73_

### Electrochemical measurements

Using emery paper grades 200–1000–2000–4000, the working electrodes (1 cm diameter) were polished to a mirror-like prior to each experiment. Before being placed in the polarization cell, the working electrodes were degreased in pure ethanol and acetone and washed in flowing bidistilled water. Electrochemical testing with a three-electrode setup was carried out using the VersaSTAT4 potentiostat. Platinum mesh, calomel electrodes, and Al–Ti alloys have been utilized as the counter, reference, and working electrodes, respectively. The working electrode’s diameter was one centimeter. Electrochemical tests began after the open circuit potential (OCP) had been stable for 30 min. Potentiodynamic polarization tests were performed using a scan rate of 1 mV/s and a voltage of ± 0.25 versus OCP. EIS was carried out using a 10 mV sinusoidal perturbation electric potential signal at 10 points per decade OCP increments at frequencies between 100 kHz and 10 mHz. For data fitting, ZsimpWin version 3.6 was utilized. All electrochemical experiments were performed between 25 and 55 ± 0.5 °C. A scanning electron microscope was used to examine the surface morphology of Al and Al–Ti alloys (FE-SEM, QUANTAFEG 250, Netherlands).

### Methods for assessing corrosion parameters

After 30 min of immersion, the investigated electrodes reached a steady-state of open-circuit corrosion potential (OCP) in the absence and presence of the investigated inhibitor. Tafel Polarization was used to calculate the corrosion current density (*i*_corr_) and (*E*_corr_) of the examined electrodes in the absence and presence of the inhibitor^21^.

The inhibition efficiency (*IE*%) is calculated from:

1 $$IE\text{\%}=\left[\left({i}_{\text{free}}-{i}_{\text{inh}}\right)/{i}_{\text{free}}\right] \times 100,$$

where *i*_free_ and *i*_inh_ denote to the corrosion current densities of uninhibited and inhibited solution, respectively.

The surface coverage degree is evaluated as follows:

2 $$\theta = \left[\left({i}_{\text{free}}-{i}_{\text{inh}}\right)/{i}_{\text{free}}\right].$$

### Details of the computation

The isolated molecules were subjected to quantum chemical computations by using Gaussian 16 software’s implementation of the B3LYP-D3/6-311++ G(2d, 2p)/B3LYP-D3/6-311(d, p) modeling chemistry^22^. Utilizing the universal solvation model, full geometry optimization of the isolated molecule has been carried out in the water phase (SMD)^23^. Frequency calculations follow this stage to determine the characteristics of the stationary points. Several quantum chemical descriptors were generated in this situation to find a correlation with the experimental results. The highest occupied molecular orbital’s energy is one of their characteristics (*E*_*HOMO*_), the lowest unoccupied molecular orbital (*E*_*LUMO*_), energy gap (Δ*E*), dipole moment (*μ*), global electronegativity (*χ*), chemical hardness (*η*), and electrophilicity index (*ω*), the fractions of electrons transferred (Δ*N*), and the total energy change (Δ*E*_T_). In the past, the literature has reported on the mathematical descriptors of these descriptors^24^.

The Adsorption Locator module, built-in Materials Studio 2017^25^, used Metropolis Monte Carlo simulations^26^ to pinpoint the most stable arrangement of the adsorbates on the aluminum surface. The Al (111) surface is the most stable of the many aluminum surfaces^27^. Consequently, we decided to use it to model the adsorption behavior. (2.9 2.9 5.9 nm) is the size of the simulation box when periodic boundary conditions are used. The metallic surface comprises five layers to guarantee adequate depth, each having 121 aluminum atoms.

By adding 5 five molecules of HCl, and 139 molecules of H_2_O to one inhibitor molecule on the surface of Al, the medium of HCl (0.5 M) was simulated. The simulation was performed utilizing the COMPASS force field^28^, and the electrostatic and energetic Van der Waals components were calculated using the Ewald and atom-based summations, correspondingly.

## Results and discussion

### Electrochemical behavior of Al and Al–Ti alloys in the absence and presence of 2-MBT

The *E*_corr_ values for Al and Al–Ti electrodes (Table 2) show that for greater titanium concentrations (alloys AT-1, AT-2, AT-3, and AT-4) compared to pure Al, *E*_corr_ moves to a more positive direction. This shift is related to a rise in the rate of corrosion in the case of 0.5 M HCl^29^. On the other hand, *E*_corr_ of Al and Al–Ti alloys in 0.5 M HCl solution comprising different concentrations of 2-MBT inhibitor at 25 °C exhibit different shifts. In the case of Al, AT-2 (except for 2-MBT shifts to a positive value in the presence of 0.001 mM), and AT-4, the change in the values of potential *E*_corr_ to negative proves that the inhibitor is a mixed type, and mainly cathodic^30^; with 2-MBT concentrations, this tendency is attributed to variations in the potential of the hydrogen evolution process toward more negative potentials. This shift may indicate that active cathodic sites are more blocked than anodic ones^31^. It is also evident from AT-1 and AT-3 alloy that the addition of inhibitor shifted corrosion potential *E*_corr_ in an anodic direction, indicating that the inhibitor’s adsorption was more successful at anodic than cathodic sites^32^. The observed variation in *E*_corr_ values has been repeatedly reported^33–35^. Specifically, if the *E*_corr_ displacement is greater than 85 mV, the inhibitor would be classified as a cathodic or anodic type inhibitor. On the other hand, if the displacement is lower than 85 mV, the inhibitor would be related as mixed type. In the current study, the maximum value of *E*_corr_ displacement was detected to be much lower than 85 mV, suggesting that 2-MBT is associated to the mixed-type inhibitor.

**Table 2:** Corrosion parameters of pure Al, AT-1, AT-2, AT-3, and AT-4 alloys after 30 min of exposure to 0.5 M HCl solution comprising different concentrations of 2-MBT inhibitor at a temperature of 25 °C.

Metal and alloys	Inhibitor conc. (mM)	*i*_corr_ (µA cm^−2^)	− *E*_corr_ (mV) (SCE)	*b*a (mV dec^−1^)	− *b*c (mV dec^−1^)	θ	IE%
Al	Blank	223.00	832.59	19.40	122.30		
	0.001	125.91	850.81	21.90	122.60	0.44	43.54
	0.01	100.53	851.52	20.30	122.10	0.55	54.92
	0.1	91.26	845.12	24.20	122.10	0.59	59.08
	1	61.80	845.87	26.20	123.20	0.72	72.29
AT-1	Blank	400.82	822.04	25.40	127.60		
	0.001	237.268	794.89	31.5	122.3	0.41	40.80
	0.01	174.047	803.125	23.2	124.3	0.57	56.58
	0.1	129.238	812.679	26.3	123.1	0.68	67.76
	1	96.792	781.908	33.4	124	0.76	75.85
AT-2	Blank	503.84	822.16	22.70	120.10		
	0.001	307.268	799.91	27.5	126.3	0.39	39.01
	0.01	225.214	843.702	22.4	123.4	0.55	55.30
	0.1	174.737	842.806	23.1	122	0.65	65.32
	1	121.64	854.796	30	121.3	0.76	75.86
AT-3	Blank	1001.99	808.65	32.20	133.10		
	0.001	724.748	788.184	29.8	122.8	0.28	27.67
	0.01	612.642	793.105	28.5	123.1	0.39	38.86
	0.1	448.302	769.451	25.3	121.9	0.55	55.26
	1	366.465	774.025	26.6	123.3	0.63	63.43
AT-4	Blank	896.17	804.36	32.5	129.8		
	0.001	452.896	826.339	24.7	123	0.49	49.46
	0.01	371.996	825.092	30.9	121.1	0.58	58.49
	0.1	294.623	839.644	24.6	121.8	0.67	67.12
	1	180.088	840.923	27	123.4	0.80	79.90

**Figure 2:**
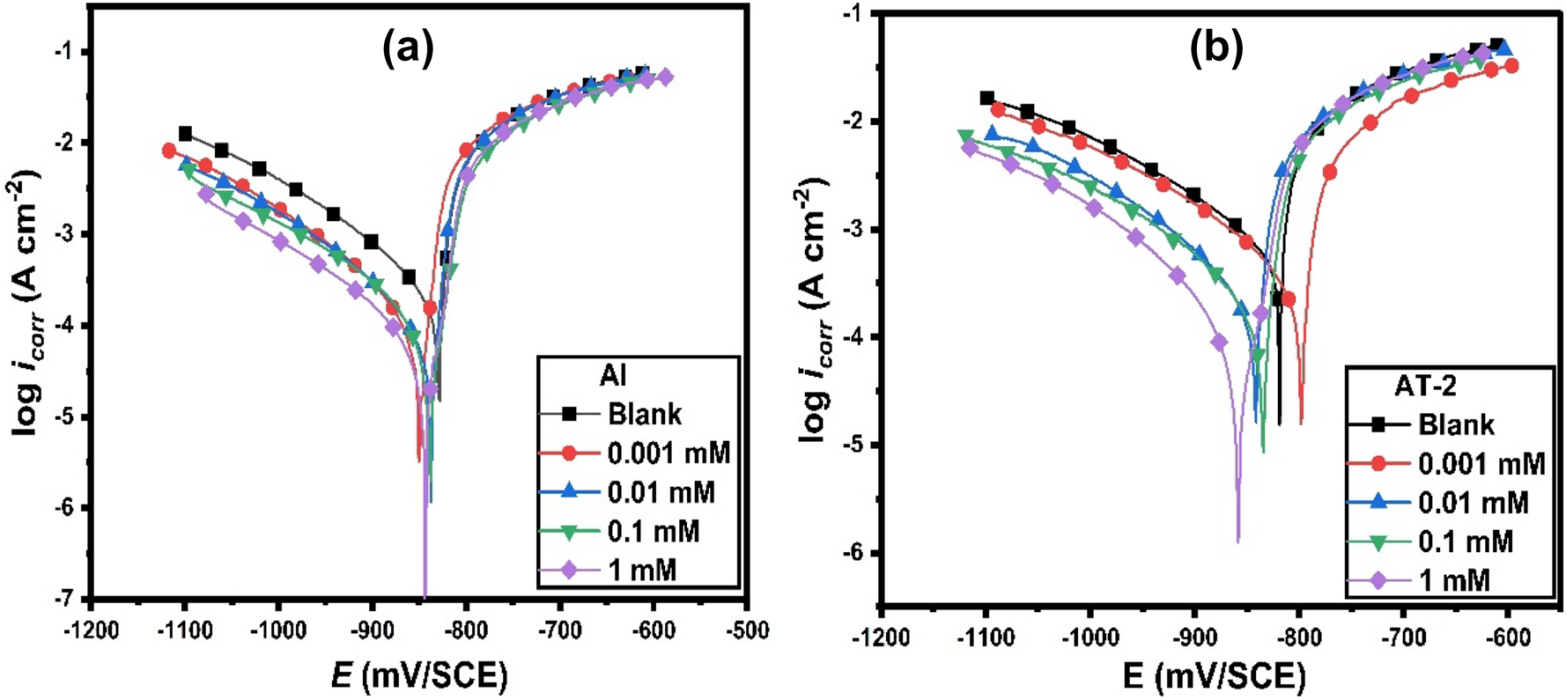
Tafel polarizations for (**a**) Al and (**b**) AT-2 alloy in 0.5 M HCl in the absence and presence of various concentrations of 2-MBT at 25 °C.

Anodic and cathodic potential against current density were used to calculate the corrosion parameters concerning the Tafel potential areas^36,37^. Figure 2a,b depicts the data obtained from the experimental curves of polarization in the presence of 2-MBT concentrations in the case of Al and AT-2 alloy. In this context, Table 2 shows the experimental results derived from the polarization curves with adding various concentrations of 2-MBT. The *i*_corr_ for the tested metal and its alloys in the presence and absence of the inhibitor has been estimated by extrapolating the cathodic and anodic lines of the Tafel polarization curve to the *E*_corr_. The presence of 2-MBT causes a decrease in both the anodic and cathodic branches of the polarization curves. However, the data exhibited that the inhibition action in the cathodic process is higher than in the anodic one. The anodic and cathodic potentials against current density were employed to compute the corrosion parameters.

Because *b*_c_ and *b*_a_ remain nearly unchanged in Table 2 compared to uninhibited solutions, the inhibitory action of 2-MBT does not alter the hydrogen evolution mechanism^38^. On the other side, it has been discovered that the cathodic Tafel slopes (*b*_c_) are greater than the anodic Tafel slopes (*b*_a_). As a result, one may hypothesize that the overall corrosion kinetics of all studied electrodes were under cathodic control^39^. Additionally, the 2-MBT is a mixed-type inhibitor (that possesses the capacity to retard both cathodic and anodic reactions). In the meantime, it influences the reaction of hydrogen evolution and the dissolution of Al and Al–Ti electrodes. This was supported by the observed shift in the *E*_corr_ value obtained in the solution, which was less than 85 mV after adding 2-MBT compared to the bare HCl solution^39^. The Al–Ti phase on the alloy surface may be responsible for this opposing impact observed at different inhibitor concentrations in contrast to the pure Al^31^.

**Figure 3:**
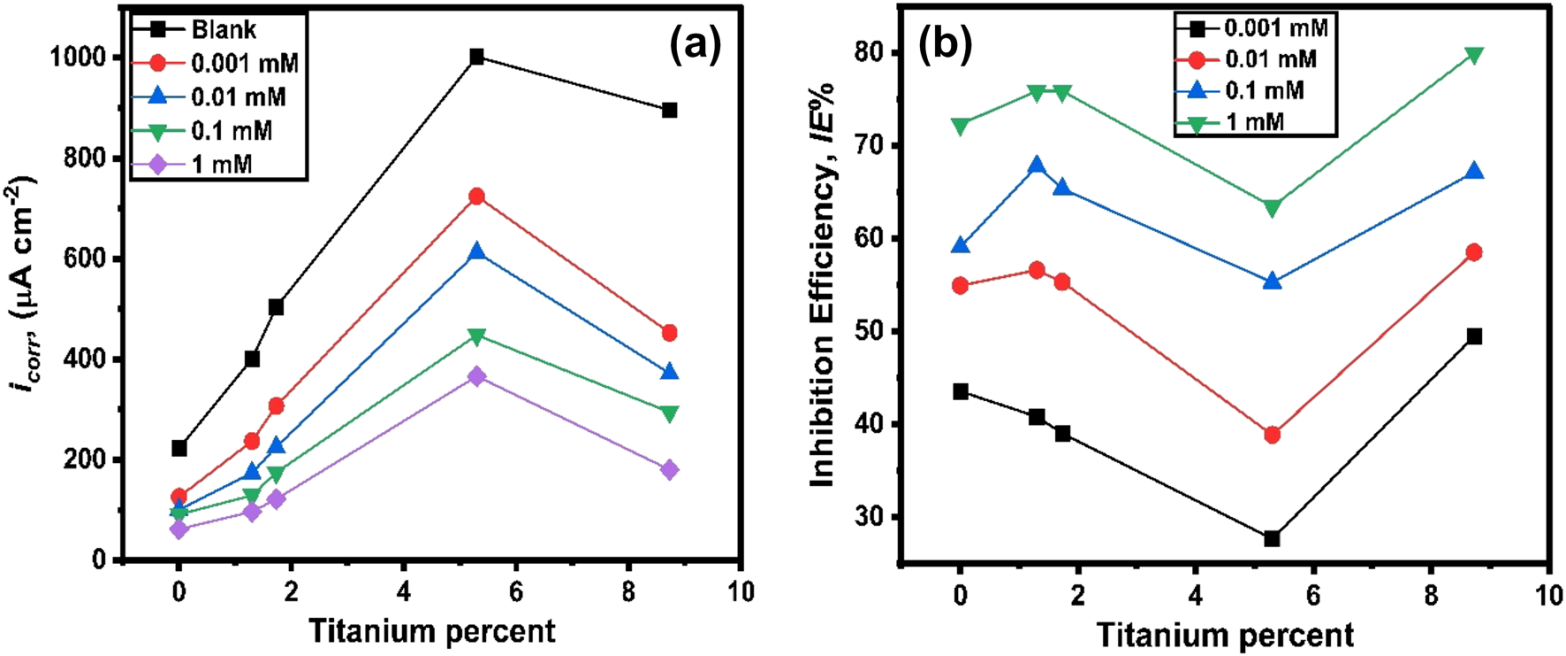
Comparison between both (**a**) *i*_corr_ and (**b**) *IE*% with titanium percent in the alloy in 0.5 M solutions of HCl containing different concentrations of 2-MBT at 25 °C.

With increasing the concentration of 2-MBT, the *i*_corr_ lowers, and the inhibition efficiency (*IE* %) of the examined electrodes increases Fig. 3a,b. For the AT-3 (5.3% Ti) alloy, the *IE* % of the tested at all the analyzed inhibitor concentrations is smaller than the equivalent value for Al and the other alloys (AT-1, AT-2, and AT-4). This effect could be explained by inhibitor molecules adsorbing is less on the mentioned alloy surface, owing to blocking active sites^40^ (see Table 2). At higher Ti content (5.3%) in AT-3, a relative drop in *IE*% value is found compared to pure Al and other alloys at various inhibitor concentrations. This is attributed to a reduction of active sites on the surface, which causes the inhibitor molecules to be less adsorbable. This is likely owing to the generation of the solid solution phase, which reduces the heterogeneity^1,41^.

However, the inhibitory efficiency values for several examined alloys (AT-1, AT-2, and AT-4) appear almost similar at high concentrations. This result could be attributed to the fact that the adsorbed inhibitor molecules mainly occupied the electrode surface of the alloys under investigation. This has led to the suppression of the vast majority of active sites. In the same context, increasing the inhibitor concentration consequently increases the coverage and adsorption amount of inhibitor molecules, resulting in a noticeable improvement in the inhibition efficiency. It is generally believed that corrosion inhibitors protect metal surfaces by adsorbing the inhibitor molecules onto the metal surfaces^42,43^.

The surface porosity percentage fraction was estimated by potentiodynamic polarization (Tafel Polarization) data. In this case, the porosity percentage (*P*_R_%) can be calculated using the following equation^44,45^:

3 $${R}_{P}= \frac{\beta }{{i}_{corr.}}\text{ and }\beta = \frac{{b}_{a}{b}_{c}}{2.303\left({b}_{a}+ {b}_{c}\right)},$$

4 $${P}_{R}= \frac{{R}_{P}^{o}}{{R}_{P}} \times 100\%,$$

where ${P}_{R}$, corresponds to the total porosity, while ${R}_{P}^{o}$ and ${R}_{P}$ are polarization resistance of the uninhibited and inhibited substrates (Al and Al–Ti alloys), respectively.

**Figure 4:**
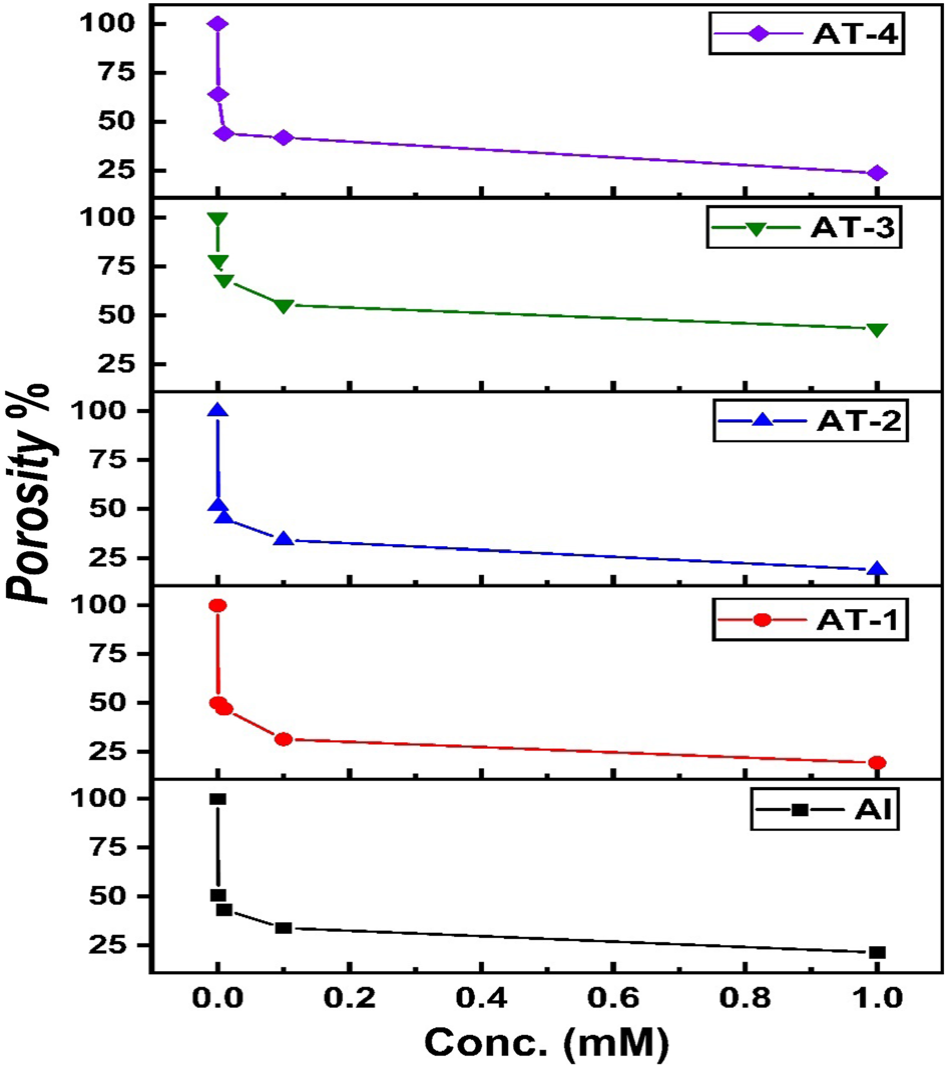
Porosity percentage as a function of 2-MBT concentrations for Al and Al–Ti alloys.

Figure 4 exhibits the porosity% variation with an increasing 2-MBT concentration with Al and its investigated alloys at 25 °C. It has been shown that the porosity% drops dramatically with increasing 2-MBT concentration. Consequently, corrosion resistance is increased in the case of inhibited substrates compared to uninhibited ones. Consequently, the observed data from Tafel plot measurements support this pattern.

### Scanning electron microscopy (SEM) micrographs

**Figure 5:**
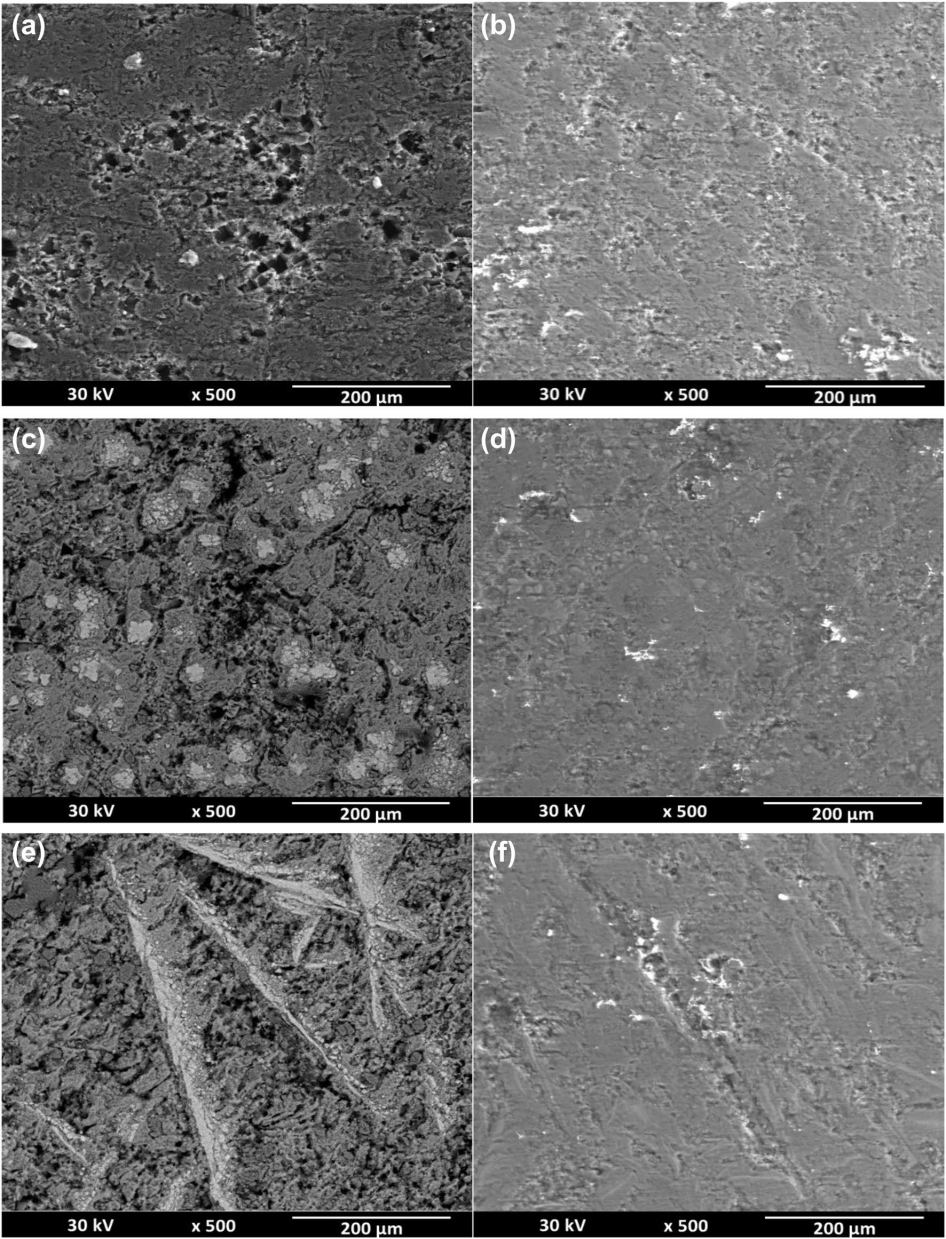
SEM micrographs of Al (**a,b**), alloy AT-1(**c,d**) and alloy AT-2 (**e,f**) after immersion for 10 h in 0.5 M HCl in the absence (**a,c,e**) and presence of 1 mM of 2-MBT (**b,d,f**) at 25 °C, respectively.

As illustrated in Fig. 5a–f, SEM images of Al, AT-1, and AT-2 surfaces were taken to determine the level of corrosion damage done in the absence and presence of inhibitor (2-MBT) on the surfaces. As a result of the extensive electrochemical corrosion caused by the immersion in 0.5 M HCl, the surfaces of the specimens had a highly rough texture with numerous deep pits on the surface. These data can be explained in terms of the activity of the chloride (Cl^−^) ion. Creating a rough surface helps to promote pit development by eliminating surface layers, allowing Cl^−^ to react directly with the metal, and consequently, the dissolving of the metal in the solution is speeded^46^. However, when exposed to the inhibitor-containing media, comparatively smooth metal surfaces with fewer pits and their depths are perceived. Therefore, examining the surface morphology indicates the formation of a protective inhibitor coating that serves as a barrier between the surfaces and the aggressive acidic environments. This adherent coating enables superior corrosion prevention of aluminum and its studied alloys, particularly when 2-MBT is present.

The porosity values obtained using the SEM micrograph are shown in Table 3. Using the ImageJ program (Java version) image processing, we assessed the uninhibited and inhibited surface porosity from SEM. It was found that the surface porosity calculated using inhibited SEM is lower than that calculated using uninhibited SEM. The decrease in porosity found in the presence of a 2-MBT inhibitor is consistent with the idea that fewer holes exist. The obtained values from Tafel polarization and SEM are nearly consistent.

**Table 3:** Porosity percentage from SEM micrographs for uninhibited and inhibited Al, AT-1 and AT-2 alloys in 0.5 M HCl without and with 1 mM of 2-MBT.

Sample	Uninhibited porosity%	Inhibited porosity%
Al	30.37 ± 1.29	10.2 ± 1.58
AT-1	35.76 ± 3.8	16.59 ± 1.82
AT-2	37.86 ± 4.6	18.33 ± 1.55

### Effect of temperature

Potentiodynamic polarization measurements for Al and its investigated alloys in 0.5 M HCl solution without and with selected concentrations of 2-MBT were evaluated at a temperature range of 25 to 55 °C to provide detailed evidence about the type of adsorption of investigated inhibitor as well as its effectiveness. The corrosion parameters revealed that raising the solution temperature rises the *i*_corr_. These results demonstrate that the reaction of cathodic hydrogen evolution and the anodic dissolution of aluminum and its corresponding alloys are enhanced by increasing temperature^47,48^. Consequently, the increase in corrosion is pronounced with the rising temperature^49^. Conversely, the slopes of the *b*_c_ and *b*_a_ Tafel lines are nearly unaltered as the temperature rises. This means that the temperature activates the corrosion of the metal surface while the corrosion mechanism remains unchanged.

**Figure 6:**
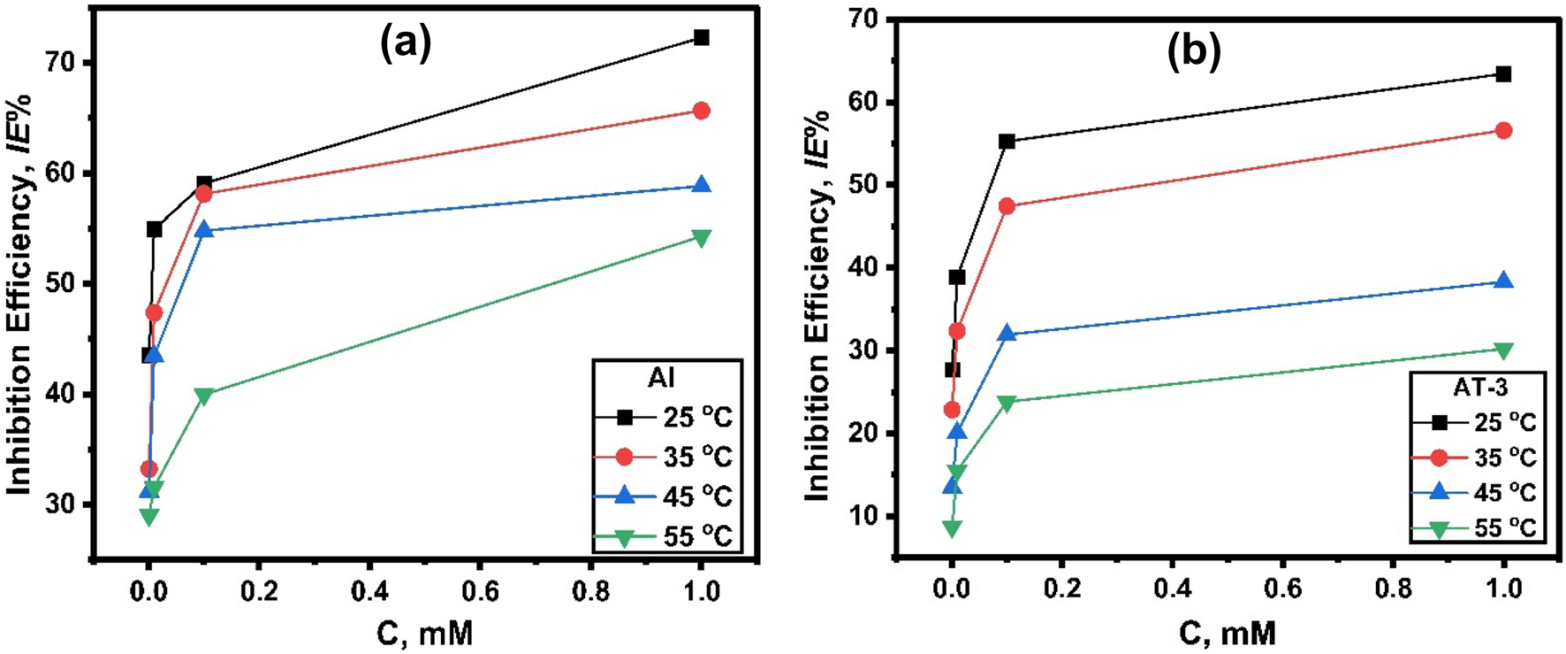
The relationship between the concentration of 2-MBT and the inhibition efficiency (*IE* %) for (**a**) Al and (**b**) AT-3 at various temperatures.

The research showed that when the temperature of Al and its investigated alloy rises, the inhibition efficiency falls. For example, Fig. 6a,b depicts the correlation between the efficiency of inhibition (*IE*%) and the 2-MBT concentration for Al and its alloys at various temperatures. This pattern could be explained by weakening the adsorption process at higher temperatures, implying that the inhibitor molecules are physically adsorbed. The adsorption of inhibitor on the electrode results in constructing a physical protective barrier, which decreases the metal reactivity in electrochemical reactions.

**Figure 7:**
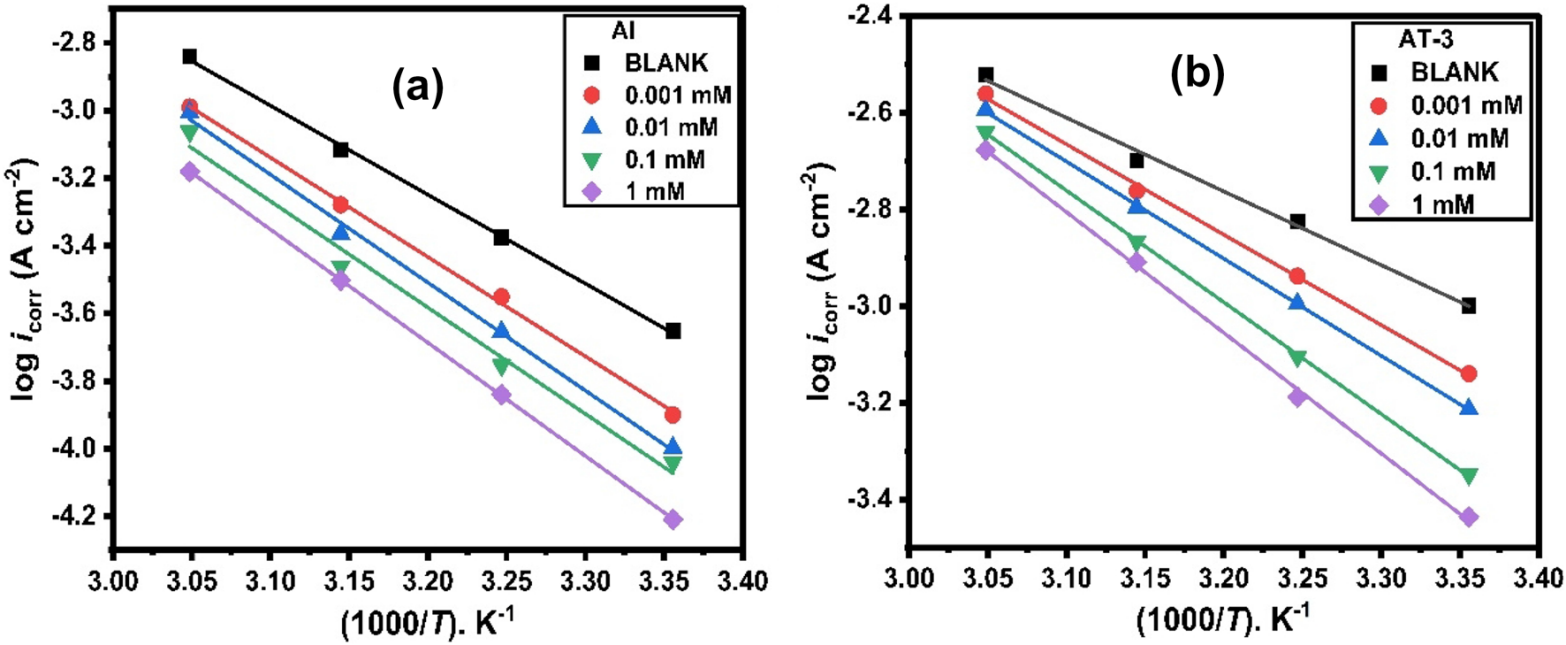
Arrhenius plots for (**a**) Al and (**b**) AT-3 alloy after 30 min of exposure to 0.5 M HCl with different concentrations of 2-MBT at different temperatures.

Figure 7a,b demonstrates Arrhenius graphs for Al and AT-3 alloy in 0.5 M HCl solution based on the presence and absence of the examined inhibitor. Arrhenius’s equation can be used to calculate activation energy^21,50,51^.

5 $${i}_{\text{corr}.}=A\text{exp}\left(\frac{-{E}_{\text{a}}}{RT}\right),$$

6 $$\text{log}{i}_{corr}=\text{log}A- \frac{{E}_{a}}{2.303RT}.$$

Associating values of *E*_a_ in the absence and presence of 2-MBT can yield significant information regarding inhibitor adsorption.

Table 4 shows the apparent activation energy (*E*_a_) for corrosion of Al and Al–Ti alloys calculated using plots of the slope of log *i*_corr_ against 1/*T*. The *E*_*a*_ values rise when inhibitor concentration increases for the investigated electrodes in 0.5 M HCl and in the presence of 2-MBT inhibitor. These findings demonstrate that the studied electrodes’ *E*_a_ values in the HCl solution are higher in the presence of 2-MBT than they are in the uninhibited acid solution. As a result, the presence of an inhibitor raises the activation energy barrier of the tested electrodes’ corrosion, reducing the corrosion rate. Due to 2-MBT’s substantial physical adsorption, the greater activation energy (*E*_a_) makes it harder to dissolve aluminum or its alloys in an acid solution^52^. An increase in the activation energy and temperature, accompanied by an additive concentration increase and an *IE*% decrease in the presence of an inhibitor, proposes that an inhibitor molecule ensures physisorption on the surface of Al and Al–Ti alloys.

**Table 4:** Activation thermodynamic parameters for Al and Al–Ti alloys in (0.5 M) HCl solution comprising different concentrations of 2-MBT inhibitor after 30 min of exposure.

Metal and alloys	Conc. (mM)	*E*_a_ (kJmol^-1^)	Δ*S*_a_ (J mol^−1^ K^−1^)	Δ*H*_a_ (kJ mol^−1^)	*E*_a_ − Δ*H*_a_ = *RT* (kJ mol^-1^)
Al	Blank	50.36	− 154.71	47.76	
	0.001	56.24	− 139.41	53.65	2.6
	0.01	61.10	− 125.33	58.50	2.6
	0.1	60.19	− 129.66	57.59	2.6
	1	64.09	− 119.17	61.49	2.6
AT-1	Blank	36.52	− 197.74	33.27	
	0.001	45.03	− 171.34	42.43	2.6
	0.01	48.91	− 160.83	46.31	2.6
	0.1	52.43	− 150.97	49.84	2.6
	1	58.26	− 134.49	55.66	2.6
AT-2	Blank	35.86	− 193.61	33.92	
	0.001	46.32	− 164.67	43.72	2.6
	0.01	50.54	− 153.14	47.95	2.6
	0.1	54.50	− 142.01	51.90	2.6
	1	58.77	− 130.80	56.18	2.6
AT-3	Blank	29.18	− 213.19	26.58	
	0.001	35.78	− 193.76	33.19	2.6
	0.01	38.44	− 186.22	35.84	2.6
	0.1	44.27	− 169.30	41.67	2.6
	1	47.80	− 159.19	45.20	2.6
AT-4	Blank	28.18	− 217.36	25.59	
	0.001	41.08	− 179.29	38.48	2.6
	0.01	44.34	− 170.71	41.74	2.6
	0.1	47.42	− 162.14	44.82	2.6
	1	59.63	− 124.93	57.03	2.6

The temperature effect was also confirmed by utilizing the Eyring transition state equation to compute the changes in activation enthalpy Δ*H*_a_ and entropy Δ*S*_a_.

7 $${i}_{\text{corr}.}= \frac{RT}{Nh}\text{exp}\left(\frac{{\Delta S}_{a}}{R}\right)\text{exp}\left(\frac{-{\Delta H}_{a}}{RT}\right).$$

**Figure 8:**
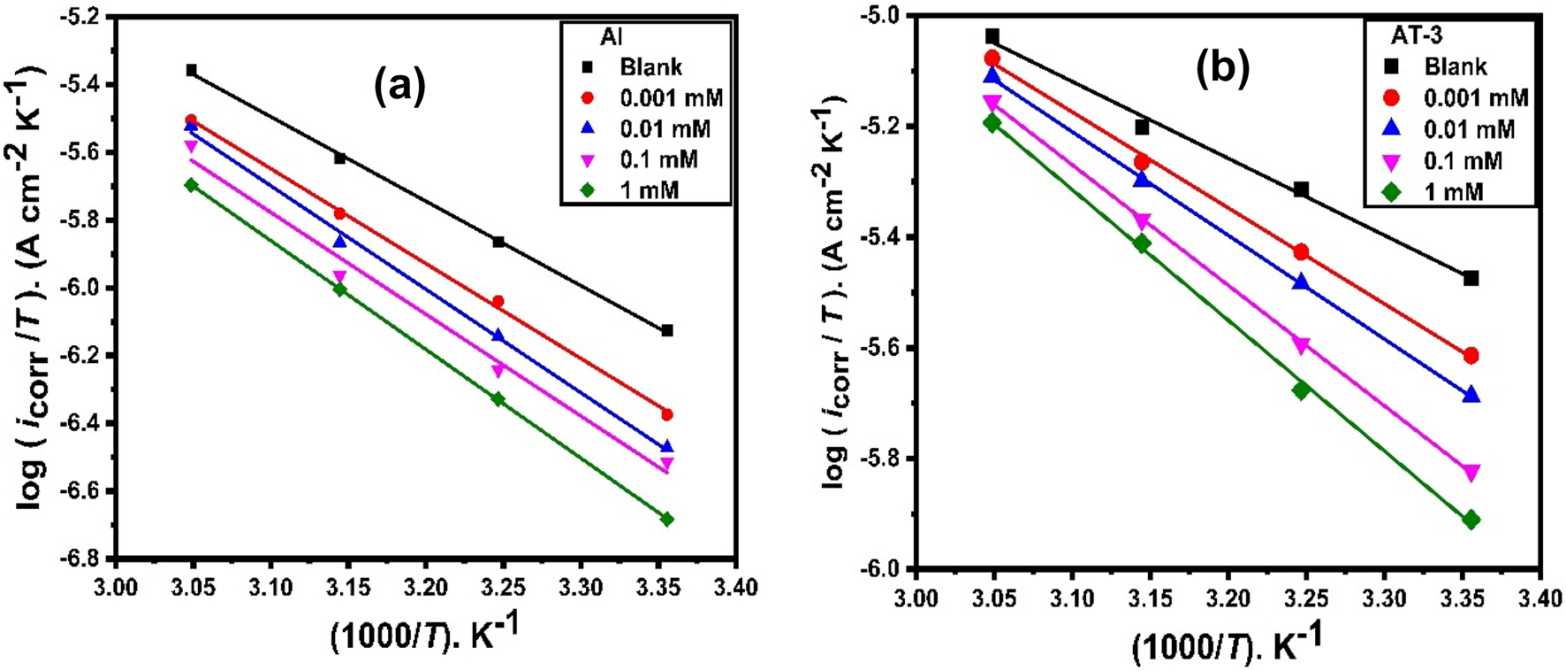
Transition state plots for (**a**) pure Al and (**b**) AT-3 alloy in HCl (0.5 M) comprising different concentrations of 2-MBT at different temperatures after 30 min exposure.

This equation can be expressed as:

8 $$\text{log}\left(\frac{{i}_{corr}}{T}\right)=\text{log}\left(\frac{R}{Nh}\right)+ \frac{{\Delta S}_{a}}{2.303R}- \frac{{\Delta H}_{a}}{2.303 RT},$$

where *h* the Planck constant, *N* the Avogadro number of the transition state plots of log (*i*_corr_/*T*) versus 1/*T* is given in Fig. 8. Δ*H*_a_ and Δ*S*_a_ were computed respectively from the slopes $- \frac{{\Delta H}_{a}}{2.303 R}$ and intercepts $\text{log}\left(\frac{R}{Nh}\right)+ \frac{{\Delta S}_{a}}{2.303R}$ of the straight lines obtained. The attained values are recorded in Table 4.

Table 4 shows that *E*_a_ and Δ*H*_a_ are both variables in the same manner. The listed results confirmed the well-known thermodynamic relationship between the two activation parameters; the following equation describes the unimolecular reactions:

9 $$\Delta {H}_{a}= {E}_{a}-RT.$$

*E*_a_ > Δ*H*_a_ by a value roughly equivalent to *RT* for 2-MBT. The activation energies (*E*_a_) are positive, and the inhibited solutions have higher activation energies than the uninhibited ones, showing that a physisorption or mix process is taking place^53^. The endothermic nature of the aluminum and Al–Ti alloys dissolution process is reflected by the positive sign of change in activation enthalpy (Δ*H*_a_). With increasing 2-MBT concentrations, the change values in activation enthalpy are increased, indicating that dissolving aluminum and its studied alloys becomes more difficult and slow^54^. The decrease in disorder from the reactant to the activated complex is indicated by the negative sign of Δ*S*_a_^55^. In 2-MBT, the activation entropy (Δ*S*_a_) change increases with increasing concentration, implying that disordering increases as it progresses from reactants to activated complex^56^.

**Figure 9:**
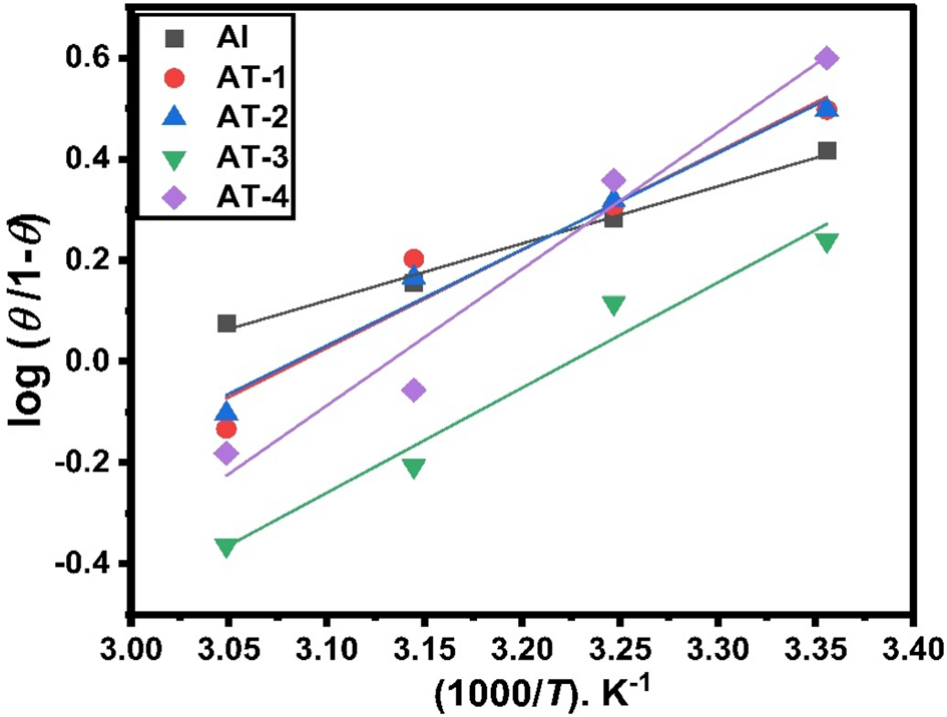
Plot of log (θ/1 − θ) against 1000/*T* for aluminum and Al–Ti alloys in 0.5 M HCl containing 1 mM of 2-MBT inhibitor.

The heat of adsorption (*Q*_ads_) was calculated using the kinetic thermodynamic model to understand better the adsorption mechanism^57^.

10 $$\text{log}\left(\frac{\theta }{1-\theta }\right)=\text{log}A+\text{log}C-\left(\frac{{Q}_{ads.}}{2.303 RT}\right),$$

where *A* is a constant, *C* is the inhibitor concentration, θ is occupied, and (1 − θ) is the vacant site not occupied by the inhibitor. The relationship between log(*θ*/1 − *θ*) and 1/*T* is shown in Fig. 9 of the electrodes under investigation in the presence of 1 mM 2-MBT inhibitor.

Negative *Q*_ads_ values correspond to physisorption properties, with inhibition efficiency decreasing as temperature rises, and positive *Q*_ads_ values indicate enhanced inhibition efficiency as temperature rises. In the acid with 2-MBT, the calculated values of *Q*_ads_ (Table 5) for aluminum and its alloys were all negative, which agrees with the postulated inhibitory physisorption properties^58^.

**Table 5:** The heat of adsorption (*Q*_ads._) for Al and Al–Ti alloys in the presence of 1 mM of MBT inhibitor.

Metal and alloys	*Q*_ads._ (kJ mol^−1^)
Al	− 21.60
AT-1	− 37.14
AT-2	− 36.41
AT-3	− 39.79
AT-4	− 51.69

### The corrosion process adsorption isotherm and the parameters of thermodynamics

Adsorption isotherms play a significant impact in understanding how organo-electrochemical systems function. For various concentrations of the organic inhibitor, the degree of surface coverage (*θ*) can be calculated using potentiodynamic polarization measurements^59^. The type of adsorption isotherm influences the surface coverage, adsorption equilibrium constant, and interaction between the organic molecule and the electrode surface. The nature of the inhibitor on the corroding surface has been determined based on its adsorption qualities during the corrosion inhibition of metals and alloys. The solvent (H_2_O) molecules are potentially adsorbed at the metal–solution contact. As a result, it is possible to conceptualize the adsorption of inhibitor molecules from aqueous solutions as a quasi-substitution process involving water molecules at the electrode surface [H_2_O_(ads.)_] and organic compounds in the aqueous phase [2-MBT_(sol.)_]^31,60^.

11 $${2{\text{-}}MBT}_{(sol.)}+ {nH}_{2}{O}_{(ads.)} \leftrightharpoons {2{\text{-}}MBT}_{(ads.)}+ {nH}_{2}{O}_{(sol.)},$$

where *n* is the number of water molecules replaced by one 2-MBT inhibitor molecule. The adsorption isotherm can provide fundamental information about the inhibitor–electrode surface interaction. To determine the isotherm of adsorption, a linear connection correlation between the surface coverage degree (*θ*) produced by Tafel polarization (*θ* = *IE%*/100) and inhibitor concentration (*C*_inh_.) is computed. Langmuir, Freundlich, Temkin, El-Awady, and Frumkin are among the isotherms that have been managed to fit the values. Langmuir isotherm provided the best fit. This isotherm implies that every possible adsorption sites are equal and that binding of particle takes place regardless of whether the surrounding sites are occupied^61^.

**Figure 10:**
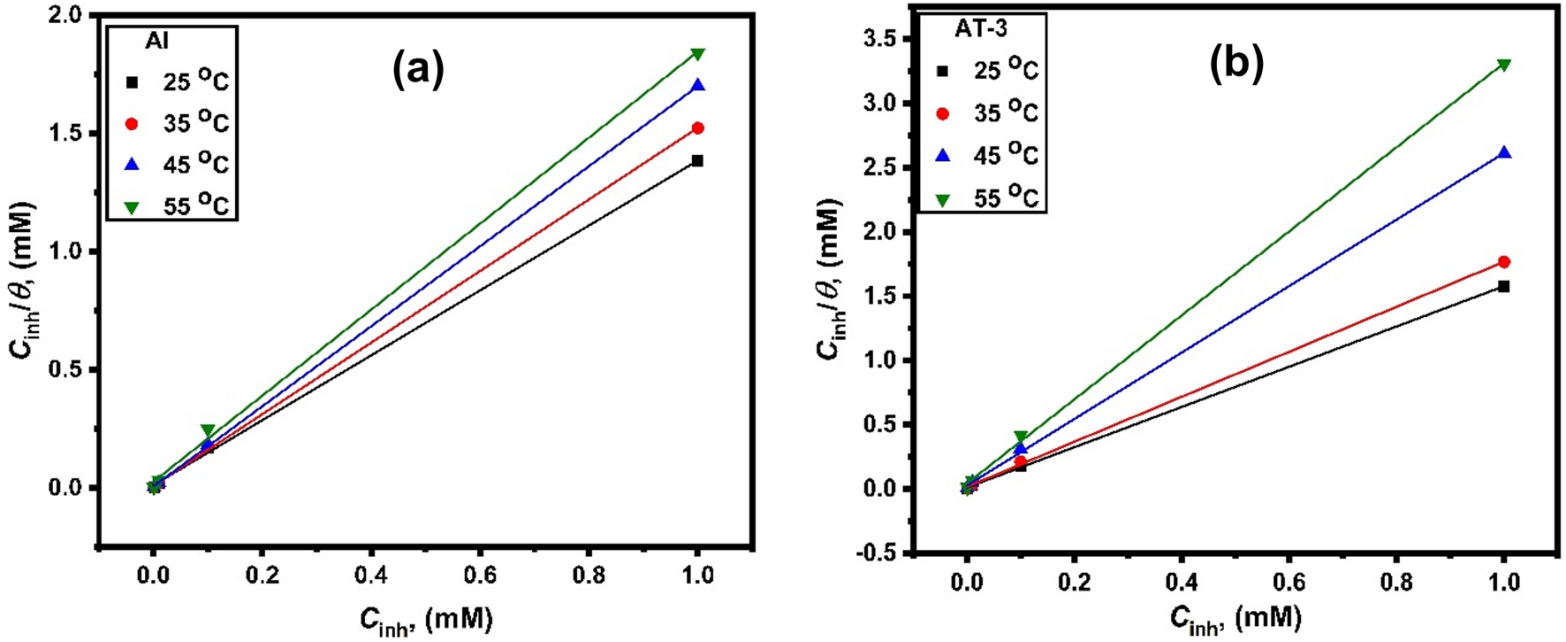
Fitting of Langmuir adsorption model (*C*_inh_/*θ* versus *C*_inh_) based on the data attained from measurements of Tafel polarization for (**a**) Al and (**b**) AT-3 alloy in 0.5 M HCl solution comprising various concentrations of 2-MBT at different temperatures.

Accordingly, *θ* is associated to *C*_inh_ using the relation:

12 $$\frac{{C}_{inh}}{\theta }= {C}_{inh}+ \frac{1}{{K}_{ads}},$$

where *K*_ads_ is the inhibitor adsorption equilibrium constant and *C*_inh_ is the inhibitor concentration. The inhibitor molecule adsorption on the surface of the electrode followed the Langmuir adsorption model, as indicated by straight lines in *C*_inh_/*θ* versus *C*_inh_ graphs (Fig. 10). The fitted curves had regression coefficients close to unity (Table 6), demonstrating the adsorption of 2-MBT molecules on Al and its alloys follows the Langmuir adsorption approach. The tested trend of the inhibitor depends on the molecule’s adsorption on the surface of the electrode^62^. The straight lines intercept with the *C*_inh_/θ axis^63^ were used to calculate *K*_ads_ values, which were then associated with the standard free energy of adsorption (${\Delta G}_{ads}^{0}$) using the following equation^64^:

13 $${K}_{ads}= \frac{1}{55.5}\text{exp}\left(-\frac{{\Delta G}_{ads}^{0}}{RT}\right).$$

**Table 6:** Thermodynamic parameters of the inhibitor adsorption on Al and Al–Ti alloys in (0.5 M) HCl solution at 25 °C.

Metal and alloys	Regression coefficient, *R*^2^	*K*_ads_, M^−1^	${\Delta G}_{ads.}^{0}$(kJ mol^−1^)	${\Delta H}_{ads.}^{0}$(kJ mol^−1^)	${\Delta S}_{ads.}^{0}$(J mol-^−1^ K^−1^)
Al	0.99934	84,745.76	− 38.06	− 16.45	72.52
AT-1	0.99984	141,843.97	− 39.34	− 15.88	78.72
AT-2	0.99969	110,987.79	− 38.73	− 37.28	4.85
AT-3	0.99978	85,106.38	− 38.08	− 35.19	9.7
AT-4	0.99954	104,821.80	− 38.59	− 8.44	101.99

In mol/L, the molar concentration of water in the solution was represented by 55.5 in the equation above^38^. The adsorption equilibrium constant (*K*_ads_; Table 6) exhibited relatively high values, indicating that this inhibitor has a large adsorption capacity on the electrode surface and, thus, a higher inhibition efficiency^65,66^.

As a general rule, the values of ${\Delta G}_{ads.}^{0}$ up to approximately − 20 kJ mol^−1^ are following the physisorption. In comparison, values around − 40 kJ mol^−1^ or higher are consistent with chemisorption, characterized by the electrons transfer from organic molecules to the metal surface, forming a coordinate bond^67,68^. The values of ${\Delta G}_{ads.}^{0}$ determined in this investigation for the examined 2-MBT inhibitor on pure aluminum and aluminum-titanium alloys in HCl (0.5 M) solution range of − 38.06 to − 39.34 kJ mol^−1^ (Table 6). The latter findings imply that the studied inhibitor adsorbs on the surface of the electrodes via both physical and chemical adsorption processes. Physical adsorption occurs due to electrostatic attraction among the inhibiting species’ dipoles or ions and the electrically charged surface of electrodes. Values of − 40 kJ mol^−1^ or more negative are consistent with charge sharing or transfer from the organic molecules to the metal surface, causing the formation of a coordinate type of bond (chemisorption)^69^. The adsorption tendency of 2-MBT on the metal surface is indicated by the negative sign of the standard free energy of adsorption, indicating that the inhibitor adsorption on the metal occurs spontaneously^70^.

According to thermodynamics, enthalpy ${\Delta H}_{ads.}^{0}$ and entropy ${\Delta S}_{ads.}^{0}$ of the adsorption process are related to ${\Delta G}_{ads.}^{0}$ by the following equations^71,72^:

14 $${\Delta G}_{ads.}^{0}= {\Delta H}_{ads.}^{0}-T{\Delta S}_{ads.}^{0},$$

15 $$\text{ln}{K}_{ads.}= -\frac{{\Delta H}_{ads.}^{0}}{RT}+ \frac{{\Delta S}_{ads.}^{0}}{R}-\text{ln}\left(55.5\right). $$

**Figure 11:**
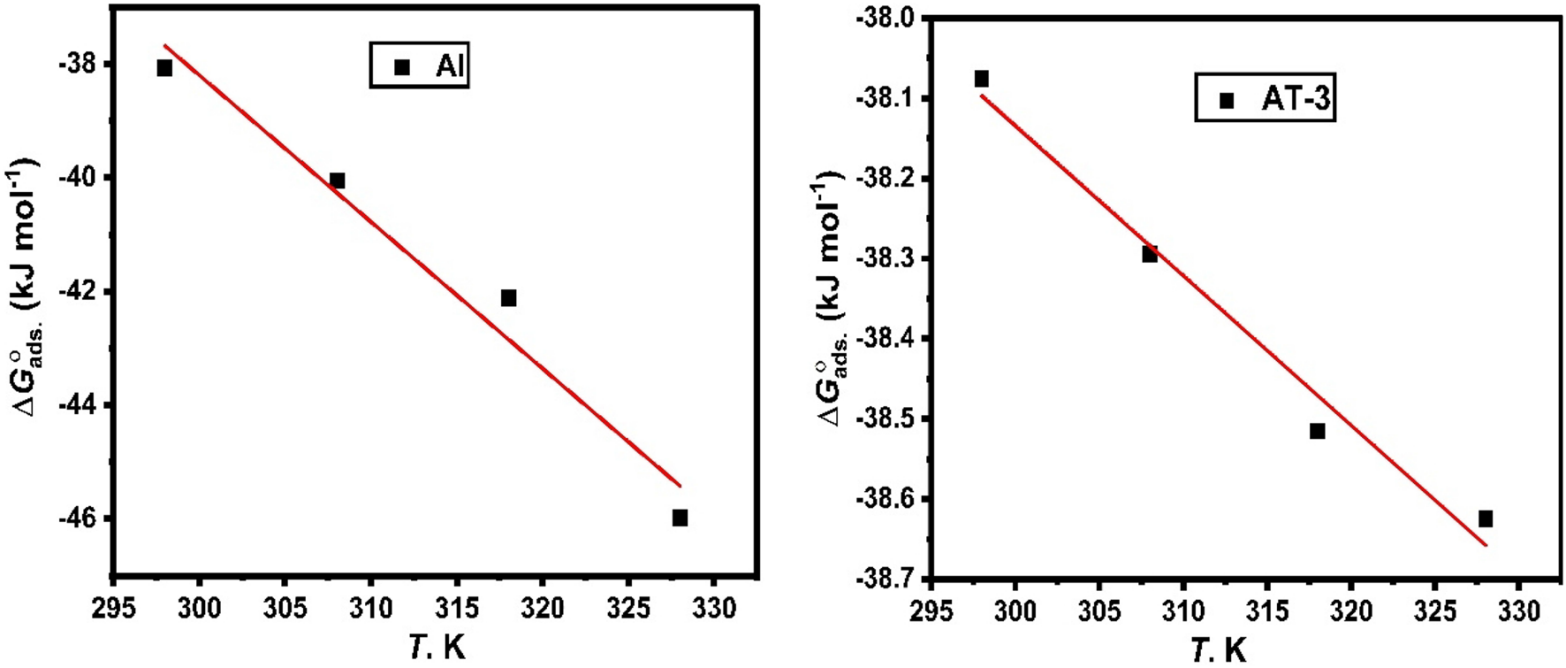
Variation of ${\Delta G}_{ads.}^{0}$ against *T* of pure Al and AT-3 alloy in 0.5 M HCl solution comprising 2-MBT inhibitor.

Figure 11 shows the plot of ${\Delta G}_{ads.}^{0}$ against *T* which provides a straight line with an intercept of ${\Delta H}_{ads.}^{0}$ and a slope of − ${\Delta S}_{ads.}^{0}$. The values of entropy and heat of adsorption estimated in Table 6 are shown. The value of $\Delta {H}_{ads}^{o}$ can provide critical information regarding an inhibitor’s adsorption process. ${\Delta H}_{ads.}^{0}$ ˂ 40 kJ mol^−1^ indicates physisorption in an exothermic adsorption process, whereas ${\Delta H}_{ads.}^{0}$ values approaching 100 kJ mol^−1^ indicate chemical adsorption^71^. Al and its alloys in the solution of 0.5 M HCl containing varying concentrations of the 2-MBT inhibitor were found to have an estimated ${\Delta H}_{ads.}^{0}$ values of − 8.44 to − 37.28 kJ mol^−1^. In accordance with the temperature-dependent variation in inhibitory efficiency, the adsorption process was exothermic associated with physisorption mechanism (Fig. 6). A negative ${\Delta H}_{ads.}^{0}$ values also indicate exothermic adsorption of inhibitor molecules^73^.

For Al and its studied alloys, the ${\Delta S}_{ads.}^{0}$ values were found to be + 4.85 to + 101.99 J mol^−1^ K^−1^. An increase in solvent energy and the water desorption entropy could explain the positive ${\Delta S}_{ads.}^{0}$ value^71^. It can also show how more water molecules can be desorbed off the metal surface by one inhibitor molecule, resulting in a rise in disorders^74^.

### Electrochemical impedance measurements (EIS)

**Figure 12:**
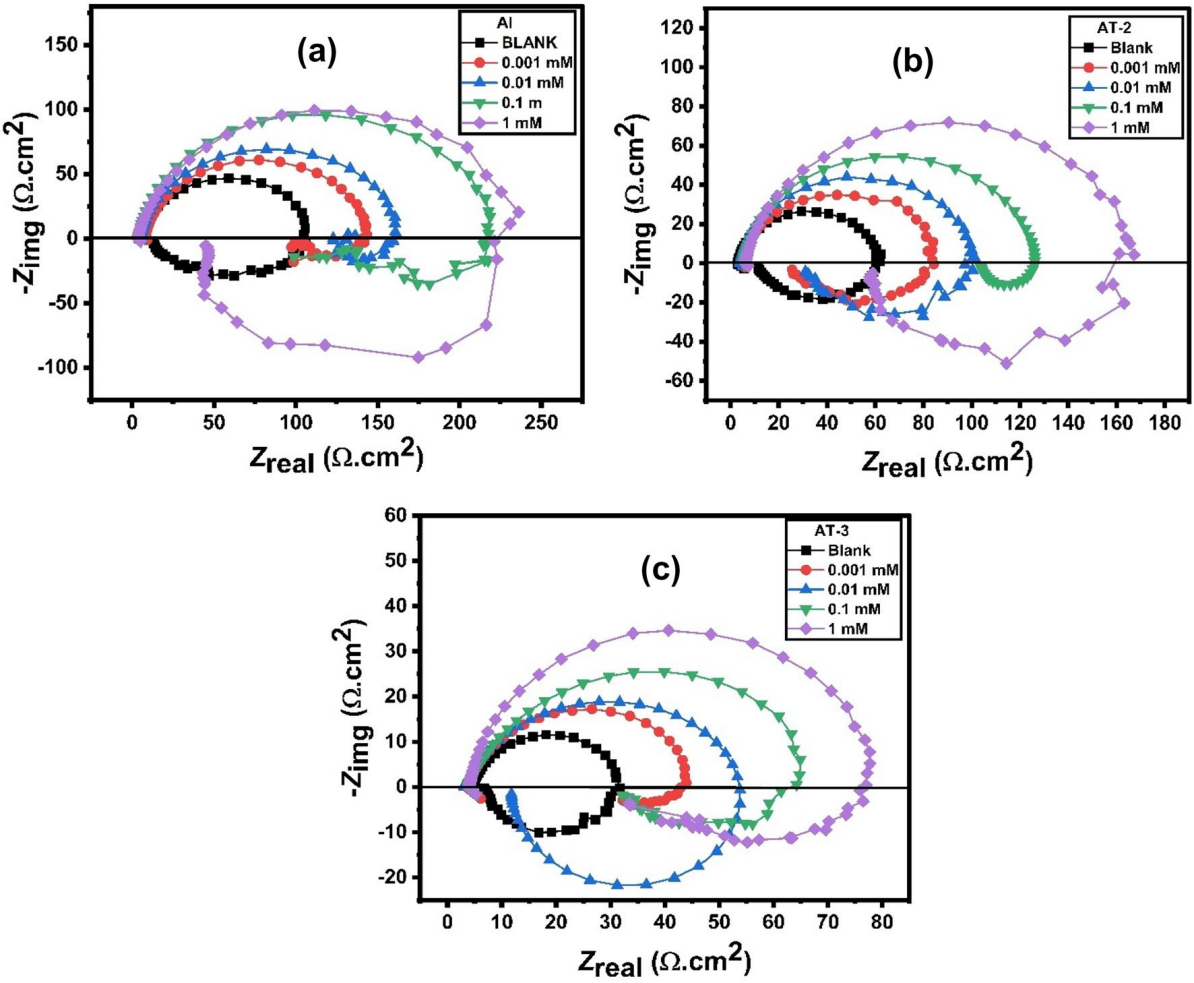
Nyquist plots for (**a**) Al, (**b**) AT-2 alloy and (**c**) AT-3 alloy in 0.5 M HCl comprising different concentrations of 2-MBT inhibitor at 25 °C.

The EIS examination in 0.5 M HCl without and with varied amounts of inhibitor was carried out to obtain information about the surface passive films on Al and its alloys (immersion time is 30 min). The Nyquist plots show a capacitive loop at high frequency (HF) and an inductive loop at low frequency (LF). Similar graphs for the corrosion of aluminum and its alloys in acidic conditions have been reported by other studies^75,76^. In Al and Al–Ti alloys, the charge transfer resistance (*R*ct) of the oxide layer may be a result of the relaxation of the H^+^ ion during the HF capacitive loop and the corrosive ions adsorption (mostly anions), such as the chloride ion onto or into the oxide film during the LF capacitive loop^77–79^. At low frequencies, the dissolution of Al or the re-oxidation of the oxide layer on the surface can also cause an inductive loop^80,81^. Surface area modulation or salt film property modification, such as density, ionic conductivity, or thickness, can be attributed to inductive activity^82^. With increasing inhibitor concentrations, the both size of HF and LF loops grew noticeably (Fig. 12). The phase angles changed to higher values as the absolute impedance’s magnitude is raised. This might be a result of the surface of the alloy producing a layer^83^.

**Figure 13:**
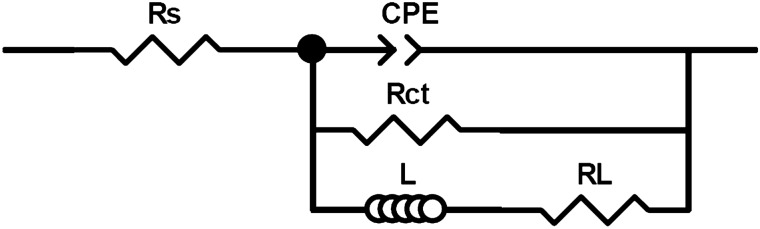
Equivalent electric circuit for quantitative estimation of EIS spectra.

In order to evaluate, the EIS data had to be fitted utilizing an equivalent electric circuit (EEC). As illustrated in Fig. 13, the most appropriate EEC was used to simulate all plots. The EEC was composed of five components: *R*_s_ represents the resistance of solution, *R*_ct_ represents the resistance of the charge-transfer, CPE represents the element of constant phase corresponding to the capacitance of double-layer (*Q*), L represents an inductive element, and *R*_L_ represents the related resistance. Because the obtained plots had depressed semicircles, CPE was used instead of actual capacitance. CPE is a term that refers to a collection of features connected with both the surface and the electroactive components. It is frequency-independent. The CPE is crucial due to the distribution of relaxation periods caused by inhomogeneities such as surface roughness/porosity, adsorption, and diffusion^78,84,85^. Moreover, the CPE contains an exponent, “*n*”, which is utilized to investigate variations in the metal/solution interface. The frequency dispersion produced by an arbitrarily distributed current on the electrode surface is responsible for the near-unity values of *n*^85,86^, demonstrating the predominant capacitive behavior^87^, as in the present study. High *R*_ct_ values are related to a slower corroding process^88^; as a result, corrosion slows down even more with higher concentrations of the investigated inhibitor aluminum and its alloys. The inhibitory efficiency values estimated using EIS correspond well with those calculated using polarization curves.

The following equation was used to get the inhibitory efficiency (*IE*%)^89^:

16 $$IE\%= \frac{{R}_{ct}-{R}_{ct}^{^\circ } }{{R}_{ct}} \times 100.$$

The values of the charge transfer resistance in the solutions with and without inhibitor are ${R}_{ct}$ and ${R}_{ct}^{^\circ }$, respectively. Table 7 shows that as the inhibitor concentration increases, the charge transfer resistance (*R*_ct._) is gradually increased and consequently the inhibitory power increases. A sluggish corroding mechanism is coupled with a high charge transfer resistance^90^. At high frequencies, there are noticeable large capacitive curves, then inductive curves at lower frequencies. The capacitive curve diameters are greater in the inhibitor solution than in the blank solution. Because the inhibitors produce an increase in the impedance, this shows that the addition of 2-MBT to the solution increases the impedance of the inhibited substrate. Li et al.^86^ proposed a similar analogy earlier. The capacitive curves are frequently linked to the corrosion process’s charge transfer. At low frequencies, the inductive curves are believed to be created by the process of relaxation, which occurs when species such as ${H}_{ads.}^{+}$ or inhibitor species that were adsorbed on the surface of the electrode^86^. As a result, the Nyquist plots’ inductive curve (Fig. 12) may be highly correlated with the presence of a passive film on Al and its alloys^91^. Various inhibitor concentrations result in bigger inductive curves than their absence, indicating a substantial involvement in the adsorption of inhibitor species onto the investigated electrodes.

**Table 7:** Electrochemical parameters and inhibition efficiencies derived from EIS for Al, AT-1, AT-2, AT-3, and AT-4 alloys in 0.5 M HCl solution comprising various concentrations of 2-MBT at 25 °C.

Metal and alloys	Conc. of inhibitor (mmol/L)	*R*_1_ (Ω cm^2^)	*CPE* (µF cm^-2^)	n	*R*_2_ (Ω cm^2^)	L (H cm^2^)	*R*_3_ (Ω cm^2^)	Ɵ	IE%
Al	Blank	5.12	59.49	0.9256	95.41	73.44	14.15		
	0.001	3.17	49.19	0.936	133.70	460.90	317.90	0.29	28.64
	0.01	4.94	82.24	0.907	159.70	465.20	538.60	0.40	40.26
	0.1	4.26	35.50	0.9433	211.22	521.20	310.20	0.55	54.83
	1	3.66	79.23	0.8897	238.00	77.89	48.38	0.60	59.91
AT-1	Blank	4.09	107.50	0.8957	80.69	47.89	43.92		
	0.001	4.03	96.74	0.9134	120.90	389.70	216.00	0.33	33.26
	0.01	4.09	42.84	0.9518	143.80	490.40	328.00	0.44	43.89
	0.1	4.26	73.67	0.9246	180.30	261.00	78.91	0.55	55.25
	1	3.92	49.95	0.9505	218.70	846.10	191.10	0.63	63.10
AT-2	Blank	3.59	69.53	0.9689	54.88	38.09	11.18		
	0.001	4.38	91.33	0.9233	77.13	87.23	37.29	0.29	28.85
	0.01	4.12	49.92	0.9408	93.91	137.90	44.28	0.42	41.56
	0.1	5.40	54.69	0.9294	122.00	413.10	505.20	0.55	55.02
	1	6.11	72.22	0.9113	158.90	176.60	79.24	0.65	65.46
AT-3	Blank	4.53	235.10	0.8895	26.21	10.57	3.28		
	0.001	4.08	542.10	0.8802	40.51	121.20	100.50	0.35	35.30
	0.01	3.06	467.20	0.785	53.58	34.37	10.32	0.51	51.08
	0.1	4.11	326.50	0.8452	61.33	71.58	64.04	0.57	57.26
	1	4.18	86.31	0.9511	72.05	126.00	77.87	0.64	63.62
AT-4	Blank	4.94	322.70	0.8624	39.84	45.66	13.39		
	0.001	4.30	84.45	0.9217	65.76	87.34	37.88	0.39	39.42
	0.01	5.37	170.00	0.8873	77.02	95.81	50.20	0.48	48.27
	0.1	5.38	179.50	0.8898	82.19	130.00	63.90	0.52	51.53
	1	5.47	65.74	0.9729	102.40	99.25	52.41	0.61	61.09

### Mechanism of inhibition of 2-MBT on Al and Al–Ti alloys

Adsorption phenomena are often impacted by the kind, the metal’s surface charge, and the organic inhibitor structure. The surface charge of the metal is caused by the electrical field that forms at the contact after immersion in the electrolyte. The location of the open circuit potential relative to the respective zero charge potential determines the surface charge of metals (PZC). The zero-charge potential is measured against a reference electrode when the metal has no charge. The ionic double layer at the electrode is nonexistent at this voltage. The electrodes can adsorb dissolved compounds in the electrolyte at zero charge potential. There is no net charge on the electrodes at PZC^92^.

**Figure 14:**
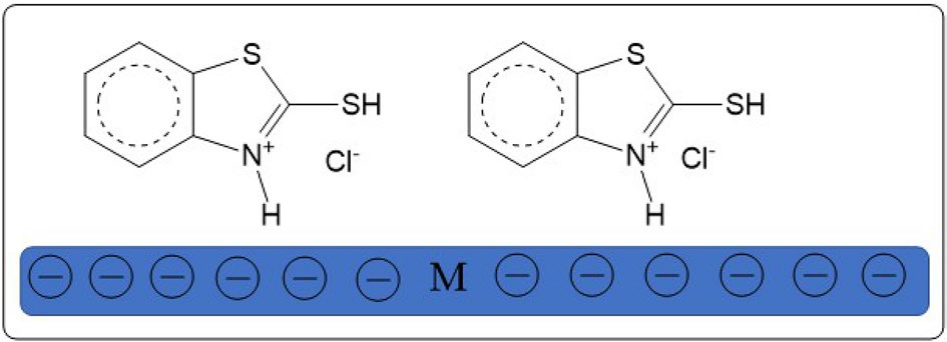
Adsorption of 2-MBT on Al and Al–Ti alloys surfaces.

The PZC of Al in 0.5 M HCl solution was determined after 30 min of exposure and was found to be − 0.4 V (vs. SHE). The values of *ϕ* potential for Al and its alloys were calculated according to the following equation^41^:

17 $$\varnothing ={E}_{corr}- {E}_{PZC},$$

where *ϕ* is Antropov’s ‘rational’ corrosion potential^93^. Hence, the values of the *ϕ* potential of Al, AT-1, AT-2, AT-3, and AT-4 are − 0.191, − 0.181, − 0.181, − 0.168, and − 0.163 V (vs. SHE), respectively. This indicates that the surface of Al and Al–Ti alloys is negatively charged at *E*_corr_. 2-MBT investigated in this study exists as protonated through nitrogen atoms (–C=N–) in HCl. The protonated inhibitor molecules could be adsorbed on investigated electrodes via electrostatic attraction, which forms between the negatively charged surfaces and protonated organic cations (Fig. 14). Adsorption involves the displacement of water molecules from the aluminum surface as well as the sharing of electrons between the hetero-atoms and metal. Furthermore, the inhibitor molecules can be adsorbed on the aluminum surface via donor–acceptor interactions between π–electrons of aromatic rings and unoccupied p-orbitals of surface aluminum atoms^94^.

### Computational study

#### DFT study

**Figure 15:**
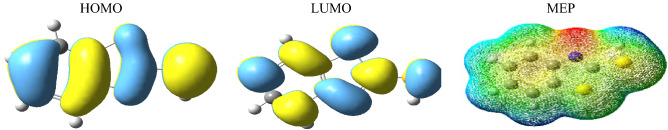
The estimated FMOs and MEP of the investigated inhibitor using B3LYP/6-311++ G(2d,2p) in the aqueous media.

Quantum chemical calculations have been primarily employed to predict the anticorrosive properties of the isolated molecule toward the metallic surface. In this respect, we calculated several global reactivity descriptors for the studied inhibitor in the aqueous phase, as illustrated in Table 8 and Fig. 15. The 2-MBT geometry was optimized at B3LYP-D3/6-311(d,p) model chemistry. This planarity of 2-MBT facilitates the horizontal loading of inhibitor molecules on the metallic surface.

**Table 8:** The B3LYP/ 6–311 +  + G(2d,2p) chemical descriptors of the studied 2-MBT in the aqueous phase.

Molecule	*E*_HOMO_, eV	*E*_LUMO_, eV	∆*E*, eV	*η*, eV	DM, D	*χ*, eV	*ω*, eV	Δ*N*, e	Δ*E*_T_, eV
2-MBT	− 6.43	− 1.36	5.07	2.54	1.44	3.90	2.99	0.54	− 0.63

Figure 15 illustrates frontier molecular orbitals’ (FMOs) electron density distribution and the molecular electrostatic potential. The findings showed that HOMO and LUMO orbitals are delocalized over the whole molecular skeleton of the investigated compound. Since the HOMO of all the studied 2-MBT inhibitor is a molecular orbital of the π-type, parallel adsorption on a metal surface is highly expected in addition to the planar geometry.

The 3D charge distribution of the molecule is shown by molecular electrostatic potential (MEP) map, Fig. 15. This map helps visualize the variably charged regions within the molecule to predict electrophilic and nucleophilic attacks on the molecule. In the MEP plot, the blue color represents the maximum positive region subject to nucleophile attack. In contrast, the red represents the negative region subject to electrophilic attack. The imine nitrogen atom of 2-MBT is observed to carry the largest electron density. Therefore, this atom will be anticipated to engage in the adsorption process on Al surface busily.

According to the frontier molecular orbital theory, the occupied orbitals of one molecule interact with the unoccupied orbitals of other species, causing attraction. In this context, the molecule with higher *E*_HOMO_ and lower *E*_LUMO_ are favorable electron-donating and electron-accepting abilities, respectively. While the *E*_LUMO_ values correlate well with the experimental results, the *E*_HOMO_ does not follow this trend. Moreover, Δ*E* is a critical parameter associated with chemical reactivity. For example, an inhibitor with a lower Δ*E* has a higher chemical reactivity increasing the number of collisions with the surface and thus increasing the chance of forming stable interactions.

The electronegativity (*χ*) of the studied molecule matches the experimental results, reflecting the discrepancy in their ability to attract the bond electrons formed with the surface. Furthermore, chemical hardness^95^ is defined as the chemical species’ resistance toward electron cloud polarization. Generally, the inhibitor molecule with a low chemical hardness value possesses high corrosion inhibition performance^96^. 2-MBT exhibits a high value of electrophilicity (Table 8), confirming its high capacity to accept electrons. The dipole moment (*DM*) is another parameter of the electronic distribution in a molecule and measures the polarity of a polar covalent bond. According to Khalil^97^, lower DM values will favor the inhibitor’s accumulation in the surface layer and, therefore, higher inhibition efficiency. Accordingly, the expected inhibition efficiency agrees with the experimental findings. The negative sign of Δ*E*_T_ indicates that the back donation’s charge transfer to the molecule is energetically favorable^98^.

#### Monte Carlo simulations

**Figure 16:**
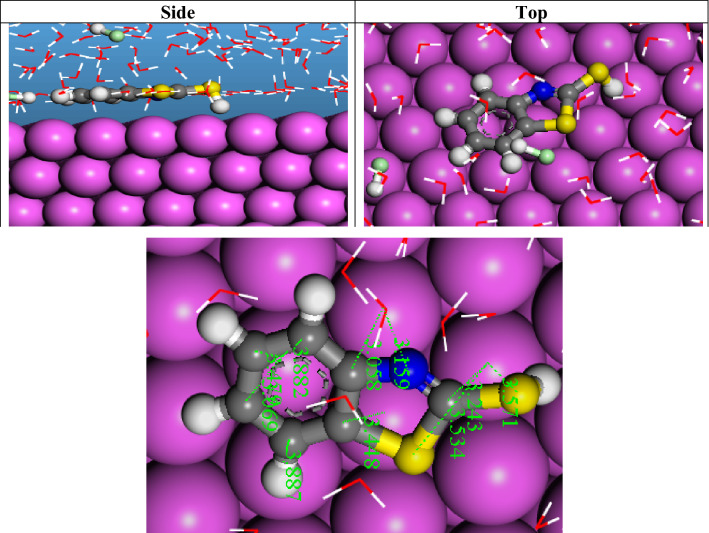
The side and top view of the most stable configurations for adsorption of 2-MBT on the Al(111) surface in an acidic medium. The below figure shows the distance data between the active atom in the inhibitor molecule and the metal surface atoms.

To minimize the contact area between the corrosion-causing materials (such as H_2_O, acidic or alkaline media) and the metal surface, molecules must parallel their structures to the metal surface as nearly as possible. This process is known as adsorption. We, therefore, sought to differentiate between the examined inhibitor’s adsorption capacity to the surface of Al(111) by doing Metropolis Monte Carlo simulations and contrasting them with pertinent empirical findings. The total energy profile for Inhibitor/Al(111) system during the simulated adsorption process by the MC approach is provided in Fig. S1. Figure 16 illustrates the best adsorption mode of the studied 2-MBT on the aluminum surface. No unusual adsorption mode on the surface was observed, where all molecules were loaded on the surface horizontally. The formation of the horizontal orientation can be ascribed to the relatively equal distribution of populations of HOMO and LUMO on the whole molecules. This result emphasizes the high adsorption strength of the studied inhibitor.

The adsorption descriptors of the investigated molecule are shown in Table 9, including total energy, adsorption energy, stiff adsorption energy, and deformation energy. In our earlier work, we have described the definitions of these parameters^99^. The most important energy parameter for adsorption is called adsorption energy, which is calculated as the total rigid adsorption energy before and after an adsorbate’s surface relaxation. According to Table 9, the negative value of the adsorption energies shows that the inhibitor spontaneously adheres to the surface of Al(111).

**Table 9:** Adsorption descriptors for Al(111)/2-MBT/(5 HCl/139 H_2_O) systems. All values are in kcal/mol.

Inh.	Total energy	Adsorption energy	Rigid adsorption energy	Deformation energy	*dE*_ad_/*dN*_i_		
					Inh	HCl	H_2_O
2-MBT	− 630	− 746	− 801	55	− 88	− 7	− 14

By taking the surface energy of Al is zero, the differential adsorption energy (d*E*_ad_/d*N*_i_) is defined as the energy required or liberated to remove a component of the adsorbate, i.e., desorption energy. The adsorption process is strongly preferred according to the inhibitor’s adsorption energy of − 746 kcal/mol and its desorption energy of − 88 kcal/mol. Despite corrosive substances like H_2_O and HCl in the media, the inhibitor preferentially adsorbs on the Al(111) surface with little to no competition because it adsorbs with much less energy.

Back to Fig. 16, the distance data between the active atom in the inhibitor molecule and the metal surface atom are depicted to judge their adsorption mode. It is shown that all distances between the inhibitor atoms and the surface ones exceed the sum of covalent radii, where they are higher than 3 Å. This supports the experimental finding that the inhibitor prefers the physical adsorption on the surface, as indicated by the activation energy and adsorption-free energy change values. It is also observed that the atoms of the imine group form relatively short physical bonds with the surface due to the high negative charge on the nitrogen atom, as indicated by the DFT results.

## Conclusion

The main conclusions deduced from this research are summarized as follows:

1. In a 0.5 M solution of HCl, the corrosion of aluminum and aluminum-titanium alloys is inhibited by 2-mercaptobenzothiazole. With an increase in inhibitor concentration, the inhibitor’s inhibition effectiveness rises.
2. At lower temperatures, 2-MBT inhibition efficiency values are higher (25 °C). However, the *IE*% values of the examined inhibitor’s Al and Al–Ti alloys at higher temperatures decrease.
3. The correlation between inhibitor concentration and the reported rise in activation energy and the decrease in the inhibitor’s inhibitory efficiency with rising temperature indicate its physical adsorption on the electrode surface.
4. Activation Thermodynamic parameters and heat of adsorption for Al and Al–Ti alloys in (0.5 M) HCl solution comprising different concentrations of 2-MBT inhibitor ${E}_{a}$ , ${\Delta H}_{a}$, ${\Delta S}_{a}$ and ${Q}_{ads}$ are evaluated and interpreted.
5. The data obtained from polarization curves fit well with the Langmuir adsorption isotherm. ${\Delta H}_{ads}^{o}$, ${\Delta S}_{ads}^{o}$ and ${\Delta G}_{ads}^{o}$ are evaluated and interpreted. The calculated values of ${\Delta H}_{ads}^{o}$ and ${\Delta G}_{ads}^{o}$ are negative, while those for ${\Delta S}_{ads}^{o}$ are positive.
6. In an acid chloride solution, 2-MBT functions as an inhibitor of mixed types of the examined aluminum and its alloys.
7. SEM and porosity percentage showed the adsorption of 2-MBT on the investigated electrodes surface and the pits on the electrode surfaces are decreased.
8. The DFT findings indicates the horizontal loading of 2-MBT on the aluminum surface.
9. The MC simulation confirms that 2-MBT prefers to protect the Al-surface through physical adsorption.

## Supplementary Information

Supplementary Figures.

## Data Availability

The datasets used and/or analyzed during the current study available from the corresponding author on reasonable request.
