# Supplementary material for: The inhibitive action of 2-mercaptobenzothiazole on the porosity of corrosion film formed on aluminum and aluminum–titanium alloys in hydrochloric acid solution
Source: Sci Rep. 2023 Mar 23;13:4812. doi: 10.1038/s41598-023-31795-2 (PMC10036543; doi:10.1038/s41598-023-31795-2)
Supplement: Supplementary file 1 — Supplementary Figures. [file 41598_2023_31795_MOESM1_ESM.pdf]

The inhibitive action of 2-mercaptobenzothiazole on the porosity of corrosion film formed on aluminum and aluminum-titanium alloy in hydrochloric acid solution.

Abdel-Rahman El-Sayed*[1], Morad M. El-Hendawy[2], Mohamed Sarwat El-Mahdy[3], Fatma S. M. Hassan[3], and Adila E. Mohamed[3].

[1]Department of Chemistry, Faculty of Science, Sohag University, Sohag 82524, Egypt.

[2] Department of Chemistry, Faculty of Science, New Valley University, Kharga 72511, Egypt.

[3]Department of Chemistry, Faculty of Science, Aswan University, Aswan 81528, Egypt.

*Corresponding author's email: elsayed777@yahoo.com

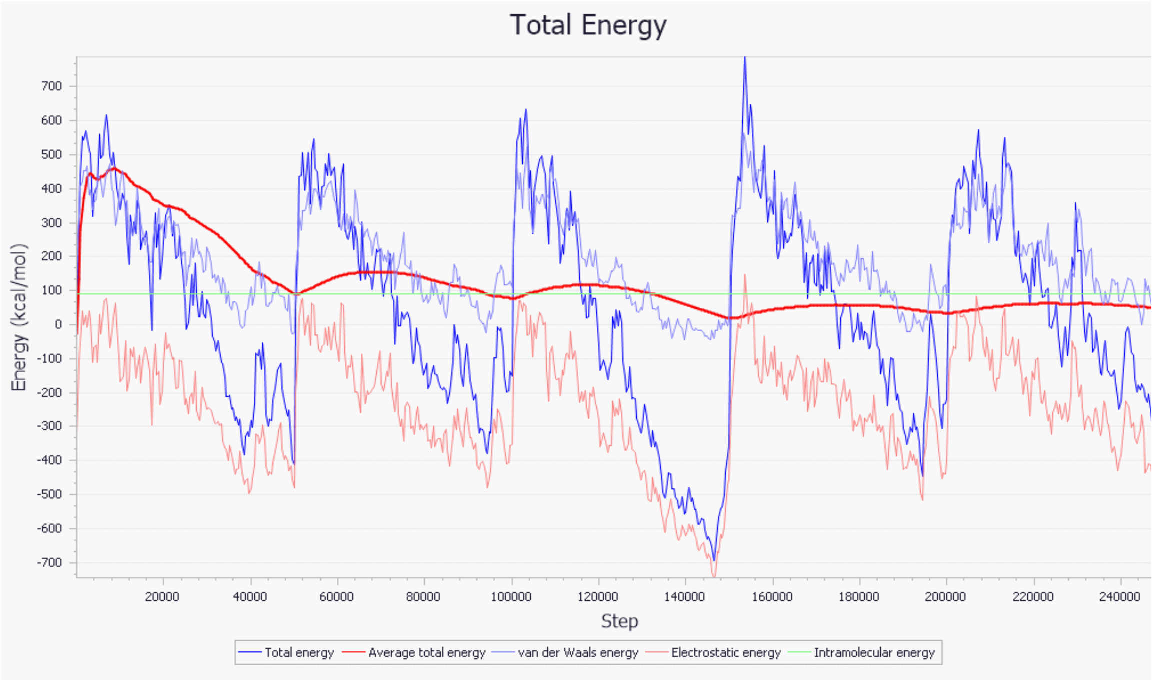

Fig. S1. Total energy profile for Inhibitor/Al(111) system during the simulated adsorption process by the MC approach.

DFT optimized structure of 2-mercaptobenzothiazole

C	0.64675800	-0.73430500	-0.00051100
C	0.81178100	0.67146300	-0.00048400
C	2.07732800	1.25860300	-0.00007400
C	3.18879800	0.41810000	0.00076700

C	3.03925700	-0.97805400	0.00048200
C	1.77747700	-1.56189800	-0.00022800
C	-1.49032600	-0.18753600	-0.00015900
H	2.19678500	2.33703500	-0.00048100
H	4.18382500	0.85222400	0.00083700
H	3.92215100	-1.60973600	0.00069500
H	1.64656100	-2.63866300	-0.00053800
S	-3.25265500	-0.33540200	0.00043500
H	-3.22471800	-1.68278900	0.00125300
N	-0.66842000	-1.18298800	-0.00077100
S	-0.76935200	1.44194000	-0.00013000

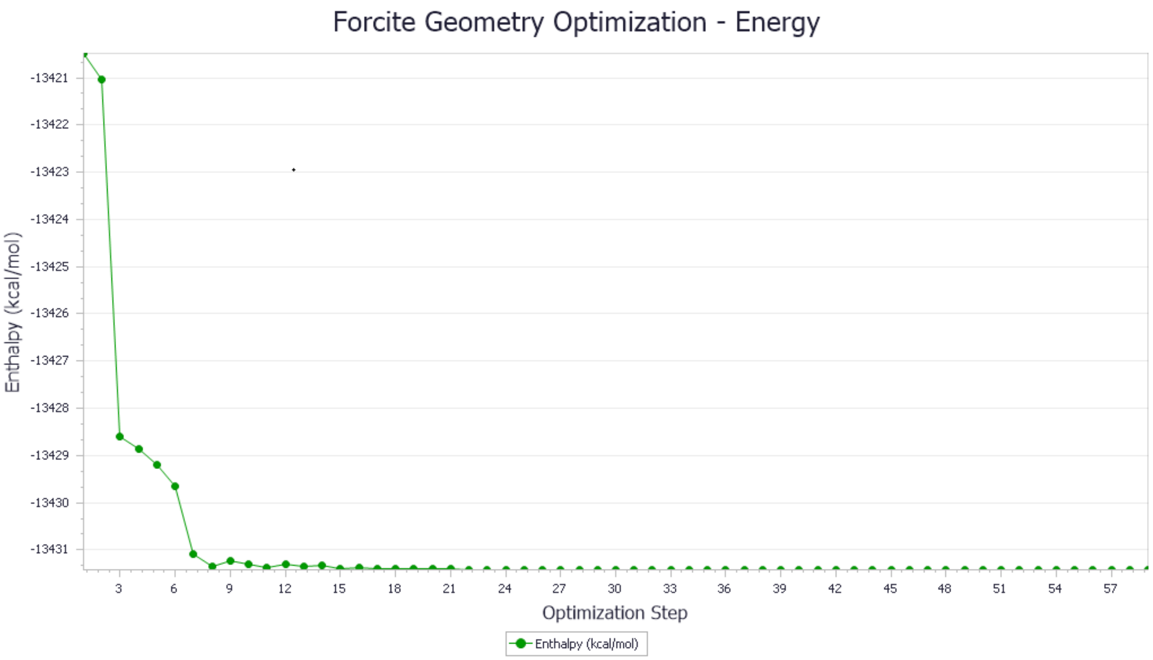

Figure S1. Geometry optimization plot (enthalpy vs. optimization step) of MC simulation for adsorption process.

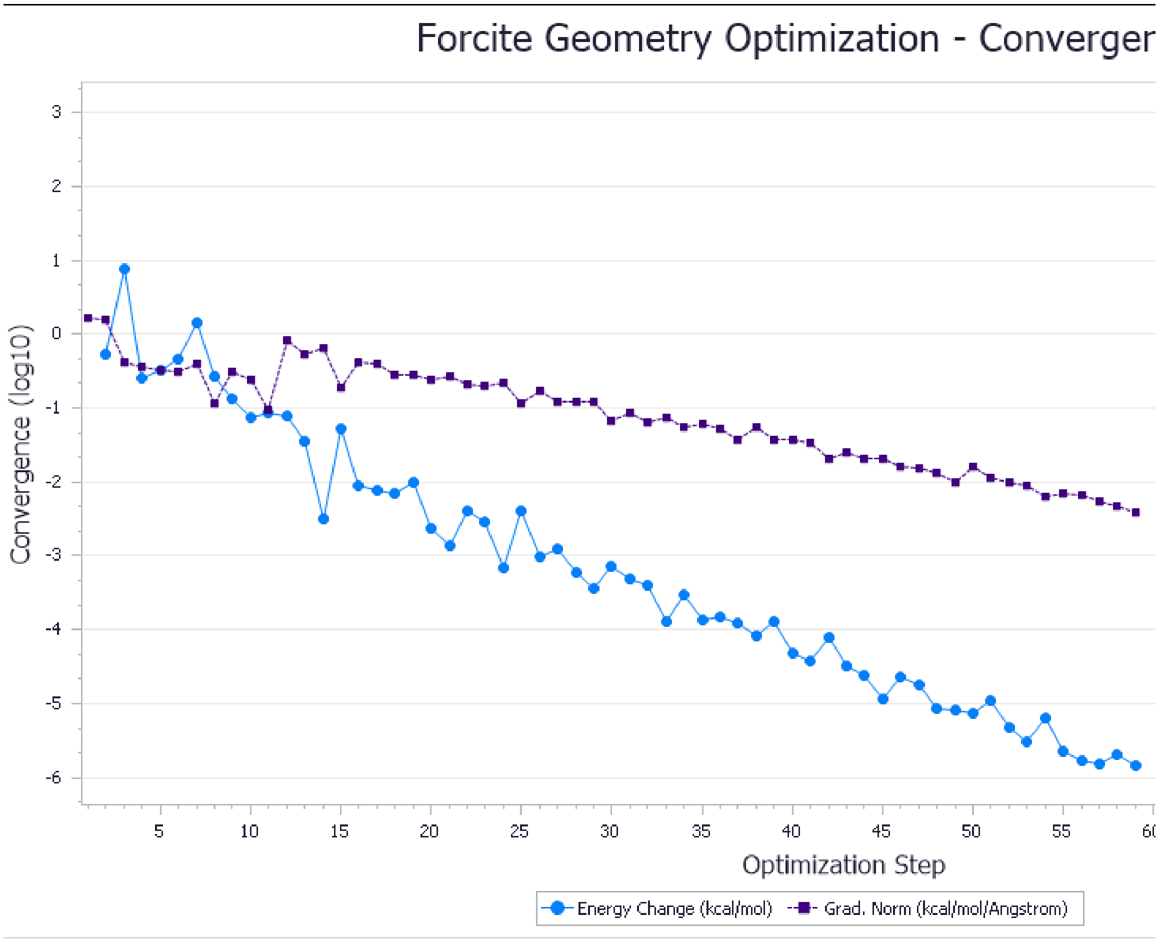

Figure S2. Geometry optimization plot (convergence vs. optimization step) of MC simulation for adsorption process.
